# SUMOylation of TEAD1 Modulates the Mechanism of Pathological Cardiac Hypertrophy

**DOI:** 10.1002/advs.202305677

**Published:** 2024-01-15

**Authors:** Xin Shi, Xuening Dang, Zhenyu Huang, Yanqiao Lu, Huan Tong, Feng Liang, Fei Zhuang, Yi Li, Zhaohua Cai, Huanhuan Huo, Zhaolei Jiang, Changqing Pan, Xia Wang, Chang Gu, Ben He

**Affiliations:** ^1^ Department of Cardiology Shanghai Chest Hospital Shanghai Jiao Tong University School of Medicine Shanghai 200030 China; ^2^ Department of Cardiovascular Surgery Shanghai Chest Hospital Shanghai Jiao Tong University School of Medicine Shanghai 200030 China; ^3^ Department of Central Laboratory Shanghai Chest Hospital Shanghai Jiao Tong University School of Medicine Shanghai 200030 China; ^4^ Department of Cardiothoracic Surgery Xinhua Hospital Affiliated to Shanghai Jiao Tong University School of Medicine Shanghai 200030 China; ^5^ General Surgery Department Shanghai Chest Hospital School of Medicine Shanghai Jiao Tong University Shanghai 200030 China; ^6^ Department of Thoracic Surgery Shanghai Chest Hospital Shanghai Jiao Tong University School of Medicine Shanghai 200030 China

**Keywords:** cardiac hypertrophy, oxidative stress, post‐translational modification, SUMOylation, TEA domain transcription factor 1

## Abstract

Pathological cardiac hypertrophy is the leading cause of heart failure and has an extremely complicated pathogenesis. TEA domain transcription factor 1 (TEAD1) is recognized as an important transcription factor that plays a key regulatory role in cardiovascular disease. This study aimed to explore the role of TEAD1 in cardiac hypertrophy and to clarify the regulatory role of small ubiquitin‐like modifier (SUMO)‐mediated modifications. First, the expression level of TEAD1 in patients with heart failure, mice, and cardiomyocytes is investigated. It is discovered that TEAD1 is modified by SUMO1 during cardiac hypertrophy and that the process of deSUMOylation is regulated by SUMO‐specific protease 1 (SENP1). Lysine 173 is an essential site for TEAD1 SUMOylation, which affects the protein stability, nuclear localization, and DNA‐binding ability of TEAD1 and enhances the interaction between TEAD1 and its transcriptional co‐activator yes‐associated protein 1 in the Hippo pathway. Finally, adeno‐associated virus serotype 9 is used to construct TEAD1 wild‐type and KR mutant mice and demonstrated that the deSUMOylation of TEAD1 markedly exacerbated cardiomyocyte enlargement in vitro and in a mouse model of cardiac hypertrophy. The results provide novel evidence that the SUMOylation of TEAD1 is a promising therapeutic strategy for hypertrophy‐related heart failure.

## Graphical abstract

Pathological cardiac hypertrophy is the leading cause of heart failure and has an extremely complicated pathogenesis. This study discovers that TEAD1 is an important transcription factor that plays a key regulatory role in cardiac hypertrophy. SUMOylation of TEAD1 is a promising therapeutic strategy for hypertrophy‐related heart failure. Mechanistically, Nrf2‐Hmox1 is a potential target of oxidative stress in cardiac hypertrophy induced by the SUMOylation of TEAD1.

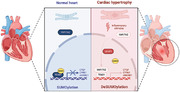

## 1 Introduction

Cardiac hypertrophy is an adaptive response of the myocardium to an increased mechanical workload resulting from adverse cardiovascular events.^[^ ^1^ ^]^ The pathogenesis of cardiac hypertrophy encompasses a complex and multifactorial process characterized by an intricate interplay between protein synthesis, cellular volume regulation, contractile function, collagen synthesis, fibrosis, inflammatory responses, and oxidative stress.^[^ ^2^, ^3^ ^]^ Pathological hypertrophy is frequently concomitant with interstitial and perivascular fibrosis, cardiomyocyte (CM) apoptosis, elevated levels of type I collagen, and myofibroblast activation.^[^ ^4^, ^5^ ^]^ Therefore, the identification of precise regulatory targets for pathological cardiac hypertrophy holds immense potential for developing innovative therapeutic strategies to address this disease.^[^ ^6^ ^]^

Members of the TEA domain family (TEAD1–4) are expressed ubiquitously in a spatial and temporal manner across all organs.^[^ ^7^, ^8^, ^9^, ^10^ ^]^ Among the four members of the TEAD family, TEAD1 has been highly conserved throughout evolution, exhibits the highest abundance in the heart, and plays a distinct role in cardiac development.^[^ ^11^ ^]^ Complete deletion of TEAD1 in the germline results in cardiac hypoplasia and embryonic lethality at E11.5.^[^ ^12^ ^]^ Moreover, embryos lacking TEAD1 and TEAD2 die at E9.5, exhibiting severe growth defects and heart tube formation abnormalities.^[^ ^13^ ^]^ TEAD1 plays a critical role in maintaining calcium homeostasis and post‐mitotic CM survival in adult CMs, and its ubiquitous or CM‐specific loss of function induces rapid‐onset severe dilated cardiomyopathy.^[^ ^14^ ^]^ A previous study showed that TEAD1 functions as a direct transcriptional regulator of protein phosphatase 1 inhibitor protein 1A in adult CMs. Notably, the loss of function of TEAD1 results in elevated PP1 activity concurrent with the reduced phosphorylation of phospholamban, ultimately culminating in diminished Ca2+ATPase 2a activity.^[^ ^10^ ^]^ As transcriptional co‐activators of TEAD1, yes‐associated protein 1 (YAP) and transcriptional coactivator with PDZ‐binding motif (TAZ) are critical for CM proliferation in perinatal cardiac development, whereas vestigial‐like family member 4 (VGLL4), a co‐repressor of TEAD1, regulates CM gene expression and inhibits early postnatal heart growth.^[^ ^15^, ^16^ ^]^ These intricate molecular mechanisms of TEAD1 contribute to the development and progression of pathological cardiac hypertrophy.

SUMOylation is a crucial post‐translational modification, facilitated by a limited set of modifying enzymes, that dynamically regulate thousands of target proteins.^[^ ^17^, ^18^, ^19^ ^]^ SUMOylation affects various biological processes as it governs subcellular localization, protein stability, and protein activity, thereby exerting regulatory control over a wide range of cellular activities.^[^ ^20^ ^]^ A previous study reported a significant decrease in the levels of small ubiquitin‐like modifier (SUMO)1 and the SUMOylation of sarcoplasmic/endoplasmic reticulum Ca2+ATPase 2a, an important protein involved in cardiac function in failing heart tissues.^[^ ^21^, ^22^, ^23^ ^]^ Thus, SUMOylation plays a pivotal role in modulating a wide range of biological processes. Uncovering novel targets that undergo site‐specific SUMOylation and elucidating their precise biological functions not only deepens our mechanistic understanding of SUMOylation in various signaling pathways but also presents exciting prospects for developing innovative therapeutic strategies for pathological cardiac hypertrophy.^[^ ^24^ ^]^

In this study, we provide the first evidence supporting TEAD1 modification by SUMO1 at lysine 173 (K173), which is reversed by SUMO‐specific protease 1 (SENP1). Notably, our findings demonstrate that during cardiac hypertrophy, the SUMOylation of TEAD1 plays a critical role in preserving protein stability, facilitating nuclear localization, and enhancing DNA‐binding ability. Moreover, this post‐translational modification promotes an augmented interaction between TEAD1 and its transcriptional co‐activator, YAP, within the Hippo pathway. We observed that deSUMOylation of TEAD1 significantly exacerbated CM enlargement both in vitro and in a transverse aortic constriction‐induced mouse model, whereas TEAD1 overexpression effectively attenuated the development of cardiac hypertrophy and remodeling. Furthermore, using RNA sequencing analysis, we propose that the SUMOylation of TEAD1 may exert regulatory control over oxidative stress responses by modulating the nuclear factor–erythroid 2–related factor 2 (Nrf2)‐heme oxygenase 1 (Hmox1) pathway. Collectively, these findings provide valuable molecular insight into the precise mechanisms through which SUMOylation governs the functional dynamics of TEAD1 during the intricate process of cardiac hypertrophy.

## 2 Results

### 2.1 TEAD1 is Elevated in CMs During Cardiac Hypertrophy

**Figure 1:**
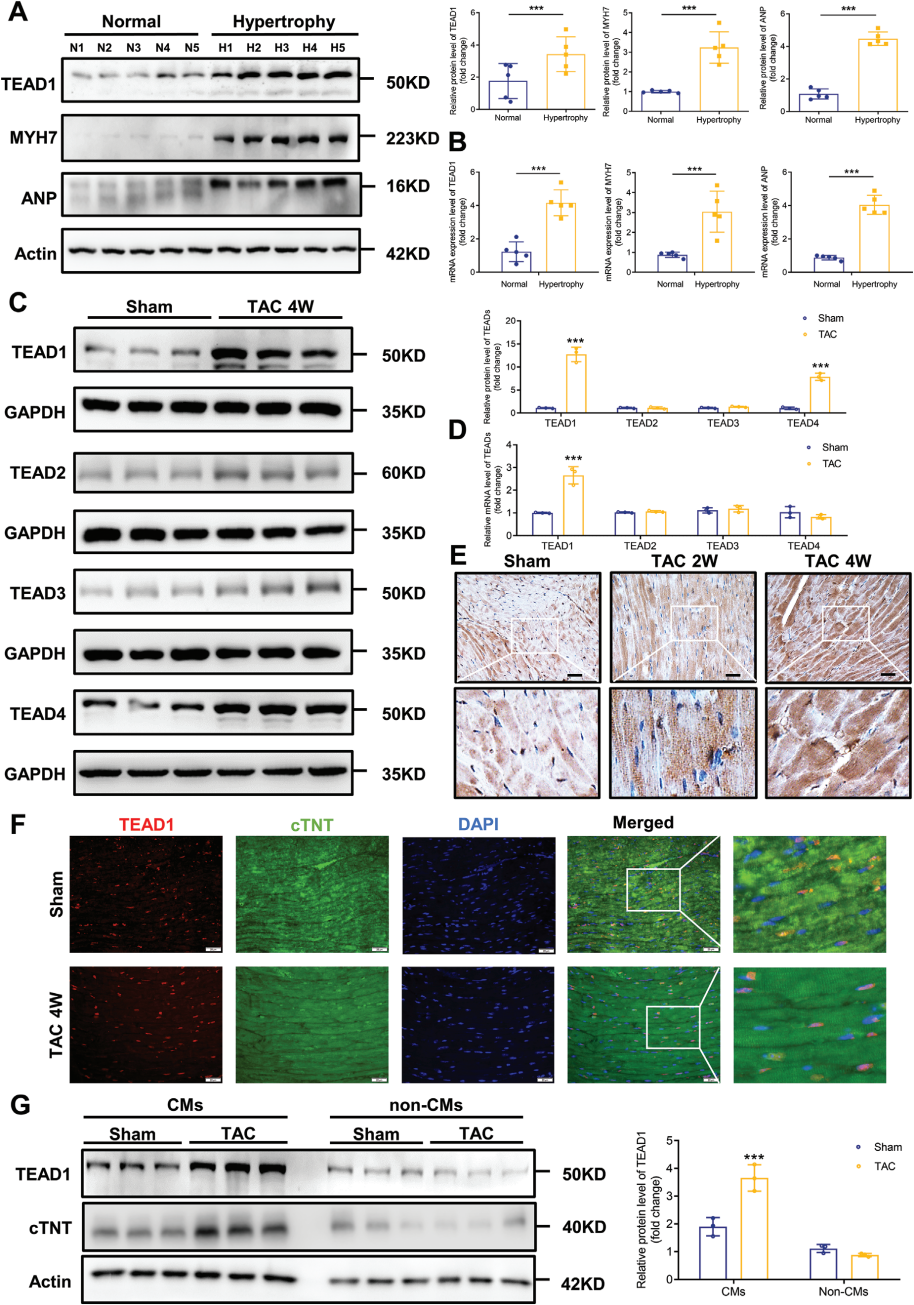
TEAD1 expression is upregulated in cardiac hypertrophy. A) Representative western blots and quantitative results of TEAD1, MYH7, and ANP protein levels in human heart tissue samples from normal donors and patients with heart failure. n = 5 per group. B) Representative RT‐qPCR and quantitative results of TEAD1, MYH7, and ANP mRNA levels in human heart tissue samples from normal donors (n = 5) and patients with heart failure (n = 5). C) Representative western blots and quantitative results of TEAD1, TEAD2, TEAD3, and TEAD4 levels in the hearts of mice subjected to transverse aortic constriction (TAC) for 4 weeks. n = 3 mice per group. D) Representative RT‐qPCR and quantitative results of TEAD1, TEAD2, TEAD3, and TEAD4 levels in hearts of mice subjected to TAC for 4 weeks. n = 3 mice per group. E) Immunohistochemistry with an anti‐TEAD1 antibody in slices from the indicated mice hearts. n = 3 mice hearts per group. F) Immunofluorescence images of TEAD1 in hearts from mice subjected to TAC for 4 weeks. Cardiac troponin T (cTNT) was used as a cardiomyocyte marker. n = 3 mice per group; scale bar, 20 µm. G) TEAD1 protein expression in cardiomyocytes and non‐cardiomyocytes from the adult mice subjected to TAC for 4 weeks. n = 3 mice per group.

To investigate whether TEAD1 expression is associated with cardiac hypertrophy, we first measured TEAD1 expression levels in heart tissues collected from five healthy controls and five patients diagnosed with hypertrophic cardiomyopathy. Immunoblot assays showed that TEAD1 protein levels were dramatically increased in patients with heart failure compared to those in healthy controls (**Figure** **1**A). Consistent with the changes in protein levels, the mRNA level of TEAD1 was also upregulated in individuals with heart failure compared to controls (Figure 1B). We further verified these results using a transverse aortic constriction (TAC) surgery‐induced hypertrophic mouse model. In this animal model, the protein expression levels of TEAD1 and TEAD4 were significantly elevated (Figure 1C). The expression levels of the other three isoforms, TEAD2, TEAD3, and TEAD4, were not markedly altered between the sham and TAC groups, while the mRNA levels of TEAD1 in the TAC groups were dramatically elevated (Figure 1D), which was further confirmed by immunohistochemistry (Figure 1E). Elevated TEAD1 was mainly localized in CMs based on its co‐localization with cardiac troponin T (Figure 1F). We compared TEAD1 expression in CMs and non‐CMs in adult mouse heart tissues. The protein expression level was markedly increased in the CM fraction, but not in the non‐CM fraction (Figure 1G).

**Figure 2:**
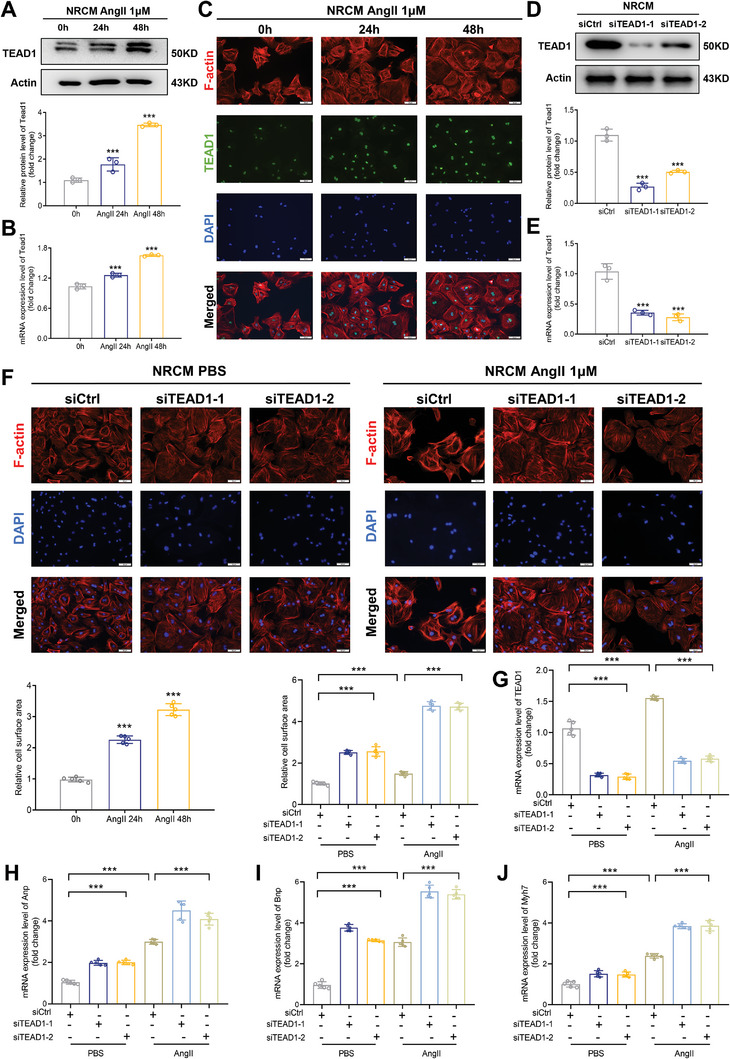
TEAD1 knockdown exacerbates angiotensin II‐induced cardiomyocyte hypertrophy in neonatal rat primary cardiomyocytes (NRCMs). A) Representative western blots and quantitative results of TEAD1 in NRCMs stimulated with or without angiotensin II (1 µm) for 24 h or 48 h. n = 3 samples per group. B) Representative RT‐qPCR and quantitative results of TEAD1 mRNA levels in NRCMs stimulated with or without angiotensin II (1 µm) for 24 h or 48 h. n = 3 samples per group. C) TEAD1 localization in the nucleus of NRCMs. NRCMs stained by F‐actin (red) and TEAD1 (green) in response to angiotensin II (1 µm) for 0, 24, and 48 h. The nuclei were stained with DAPI (blue). n = 3 per group; scale bar, 50 µm. D) Representative western blots and quantitative results of TEAD1 in NRCMs transduced with siTEAD1–1, siTEAD1–2, or siControl. n = 3 samples per group. E) Relative mRNA levels of TEAD1 in NRCMs transduced with siTEAD1–1, siTEAD1–2, or siControl. n = 3 samples per group. F) Representative immunofluorescence images of F‐actin (red) staining of NRCMs infected with siTEAD1–1, siTEAD1–2, or siControl. The nuclei were stained with DAPI (blue). Scale bar, 50 µm. G–J) Relative mRNA levels of the indicated genes in NRCMs transduced with siTEAD1–1, siTEAD1–2, or siControl incubated with or without angiotensin II (1 µm) for 48 h. *n* = 3 samples per group.

To directly investigate the role of TEAD1 in CM enlargement, we isolated neonatal rat primary CMs (NRCMs) and observed that the mRNA and protein expression levels of TEAD1 increased in response to angiotensin (Ang) II treatment (**Figure** **2**A,B). TEAD1 was localized in the nuclei of NRCMs stained with F‐actin (red) and TEAD1 (green) in response to Ang II (Figure 2C). We constructed siRNAs targeting TEAD1 (siTEAD1) to transfect NRCMs (Figure 2D,E). Compared with the negative controls, the cell surface area of CMs was markedly increased in the siTEAD1 cell group, whereas siTEAD1 notably aggravated the Ang II‐induced enlargement of the cellular surface area of CMs (Figure 2F). Similar results were observed in AC16 cells (Figures S1–S3, Supporting Information). The mRNA levels of TEAD1 in the different treatment groups were confirmed using RT‐qPCR (Figure 2G). Biomarkers of cardiac hypertrophy, such as Anp, Bnp, and Myh7, were also significantly increased in the siTEAD1 group compared to those in the siControl group (Figure 2H–J). These results indicate that TEAD1 plays a protective role against CM enlargement in vitro.

### 2.2 SUMO Modification of TEAD1 in Cardiac Hypertrophy

**Figure 3:**
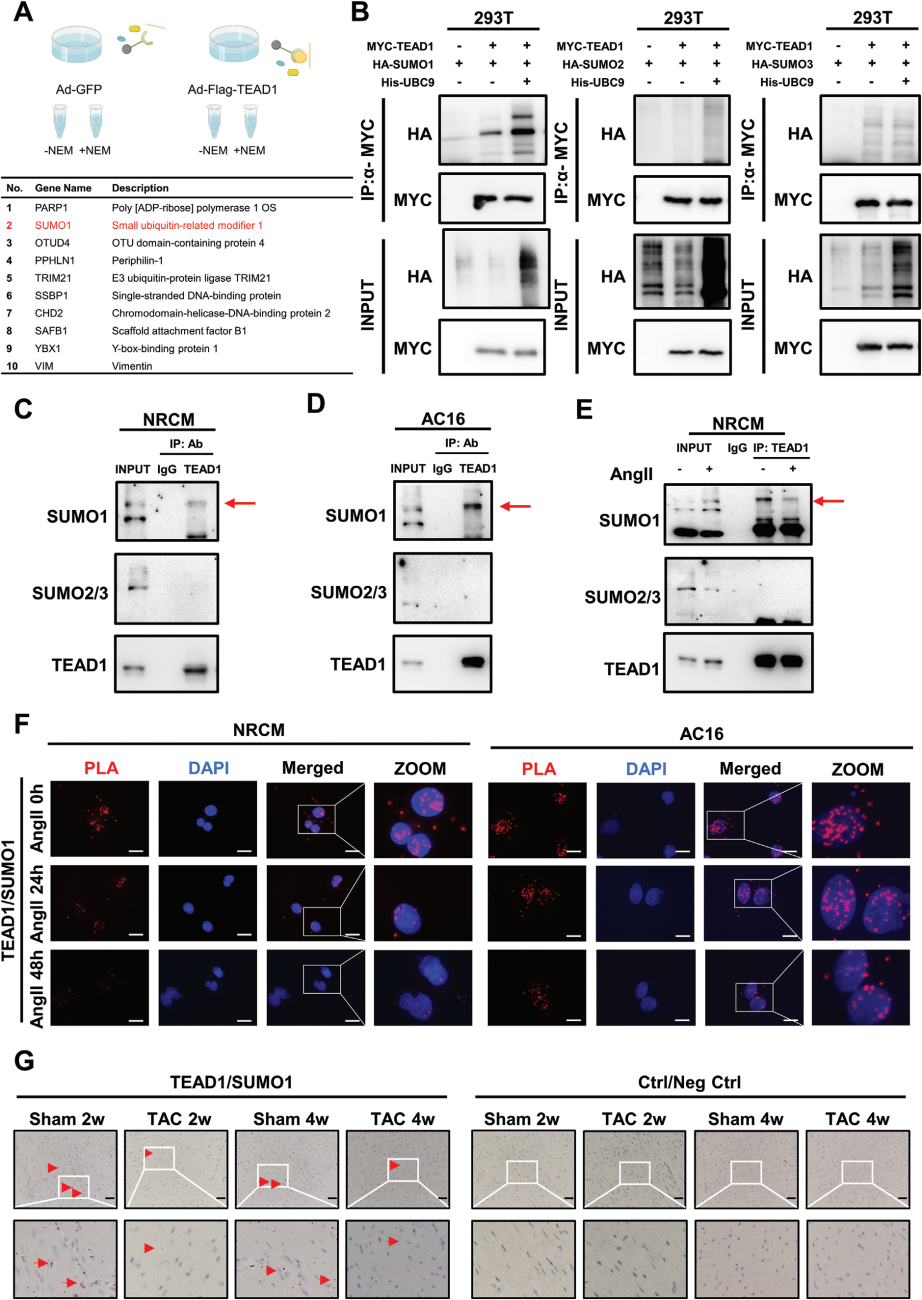
Characterization of SUMO modification of TEAD1 in cardiac hypertrophy. A) LC/MS (mass spectrometry) analysis in HEK293T cells transduced with adenoviral FLAG‐TEAD1 (Ad‐FLAG‐TEAD1) or adenoviral green fluorescent protein (Ad‐GFP) to identify the specific target of TEAD1 with or without NEM (N‐ethylmaleimide). B) Representative blots of exogenous SUMOylated TEAD1 in 293T cells transfected with HA‐SUMO1, HA‐SUMO2, or HA‐SUMO3 and MYC‐TEAD1 and His‐UBC9 in HEK293T cells. MYC‐TEAD1 was immunoprecipitated followed by immunoblotting to detect SUMO1, SUMO2, or SUMO3 (HA) and TEAD1 (MYC). C–E) Representative blots of endogenous SUMOylated TEAD1 in NRCMs and AC16 cells with or without angiotensin II. Immunoprecipitated TEAD1 was immunoblotted to detect SUMO1, and SUMO2/3. F) Proximity ligation assay (PLA) of the endogenous TEAD1 and SUMO1 or SUMO2/3 in NRCMs and AC16 cells after treatment with angiotensin II (1 µm) for 0, 24, and 48 h. Scale bar, 10 µm. (G) Brightfield PLA of the endogenous TEAD1 and SUMO1 in heart tissues of mice subjected to TAC for 2 or 4 weeks. *n* = 3 mice per group. Scale bar, 20 µm.

Post‐translational modifications, such as palmitoylation and ubiquitination are strongly associated with the function and activity of TEAD1. However, the role of SUMOylation in TEAD1 has not yet been elucidated.^[^ ^25^ ^]^ TEAD1 proteins were immunoprecipitated from the denatured lysates, with or without N‐ethylmaleimide (NEM), of human embryonic kidney (HEK)293T cells transduced with adenoviral Flag‐TEAD1 (Ad‐Flag‐TEAD1) or adenoviral green fluorescent protein (Ad‐GFP) and analyzed using liquid chromatography–tandem mass spectrometry. We identified SUMO1 among the TEAD1 Flag‐immunoprecipitated proteins (**Figure** **3**A; Table S1, Supporting Information). To confirm the SUMO modification of TEAD1, we transfected the plasmids hemagglutinin (HA)‐SUMO1, HA‐SUMO2, HA‐SUMO3, MYC‐TEAD1, and histidine (His)‐ubiquitin‐conjugating enzyme 9 (UBC9) into HEK293T cells. Interestingly, when HA‐SUMO1 was co‐overexpressed, there was a shift in the TEAD1 band in both the input and immunoprecipitation (IP) samples, indicating that a portion of TEAD1 was modified by SUMO1. In addition, co‐expression of UBC9, a SUMO E2 enzyme, enhanced the SUMOylation of TEAD1 (Figure 3B). More importantly, endogenous TEAD1 SUMOylation was detected in NRCM and AC16 cells using IP assays (Figure 3C,D). To preliminarily elucidate the pathophysiology of TEAD1 SUMOylation in cardiac hypertrophy, NRCMs were treated with Ang II, and TEAD1 SUMOylation was detected using IP assays. TEAD1 SUMOylation decreased after Ang II stimulation (Figure 3E). Immunofluorescence staining showed that endogenous SUMO1 and SUMO2/3 co‐localized with endogenous TEAD1 in NRCM and AC16 cells, respectively (Figure S4, Supporting Information). To visualize the intensity of SUMOylated TEAD1, we performed proximity ligation assays (PLAs) in NRCM and AC16 cells. The PLA signal was significantly weaker in Ang II‐treated cells (Figure 3F). In addition, we further confirmed that TAC‐induced cardiac hypertrophy in mice limits TEAD1 SUMOylation in vivo using bright‐field PLA (Figure 3G). These results suggest that SUMO1 is covalently conjugated to TEAD1 in CMs, both in vitro and in vivo.

### 2.3 SENP1 Is the Key deSUMOylase That Mediates TEAD1 deSUMOylation

**Figure 4:**
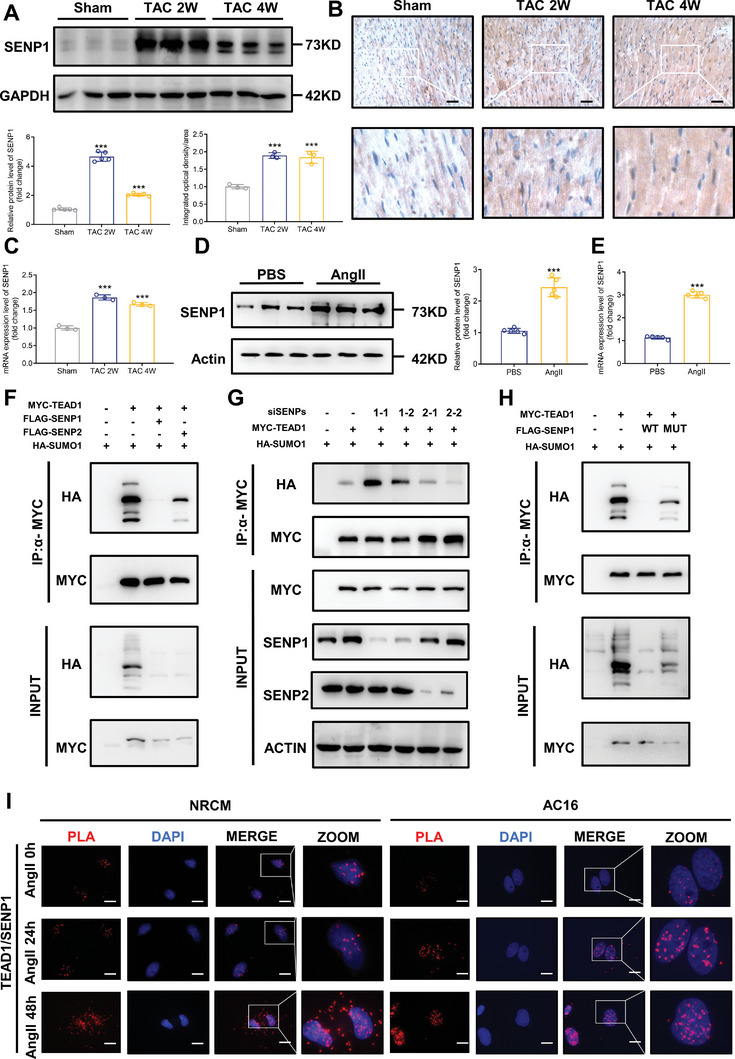
SENP1 is the key deSUMOylase that mediates TEAD1 deSUMOylation. A) Representative western blots and quantitative results of SENP1 levels in the hearts of mice subjected to TAC for 2 or 4 weeks. n = 3 mice per group. B) Immunohistochemistry with an anti‐SENP1 antibody in slices from the indicated mouse hearts. n = 3 mouse hearts per group. C) Representative RT‐qPCR and quantitative results of SENP1 mRNA levels in the hearts of mice subjected to TAC for 2 or 4 weeks. n = 3 mice per group. D) Representative western blots and quantitative results of SENP1 in NRCMs stimulated with or without angiotensin II (1 µm) for 48 h. *n* = 3 samples per group. E) Representative RT‐qPCR and quantitative results of SENP1 mRNA levels in NRCMs stimulated with or without angiotensin II (1 µm) for 48 h. n = 3 samples per group. F) Representative blots of exogenous SUMOylated TEAD1 in 293T cells transfected with FLAG‐SENP1 or FLAG‐SENP2, and HA‐SUMO1 and MYC‐TEAD1 in HEK293T cells. MYC‐TEAD1 was immunoprecipitated followed by immunoblotting to detect SUMO1 (HA) and TEAD1 (MYC). G) siSENP1–1, siSENP1–2, siSENP2–1, and siSENP2–2 in HEK293T cells were transfected with the indicated plasmids expressing HA‐SUMO1 and MYC‐TEAD1. MYC‐TEAD1 was immunoprecipitated followed by immunoblotting to detect SUMO1 (HA), TEAD1 (MYC), and SENP1. H) Representative blots of HEK293T cells transfected with the indicated plasmids expressing FLAG‐SENP1‐WT and FLAG‐SENP‐MUT (a deSUMOylation‐inactive mutant), and HA‐SUMO1 and MYC‐TEAD1. MYC‐TEAD1 was immunoprecipitated followed by immunoblotting to detect SUMO1 (HA) and TEAD1 (MYC). I) Proximity ligation assay (PLA) of the endogenous TEAD1 and SENP1 in NRCMs and AC16 cells with or without angiotensin II treatment (1 µm) for 48 h. Scale bar, 10 µm.

Recent studies have elucidated the role of SENP1 in cardiac hypertrophy.^[^ ^26^ ^]^ The expression of SENP1 is dysregulated in hypertrophic hearts, exerting a profound effect on cardiac remodeling.^[^ ^27^ ^]^ To determine whether the expression of SENP1 is associated with cardiac hypertrophy, we measured the mRNA and protein expression levels of SENP1 in a TAC surgery‐induced hypertrophic mouse model and in NRCMs treated with Ang II. The results suggested that SENP1 is upregulated at the protein level in mouse TAC‐induced hypertrophic hearts (**Figure** **4**A). To validate this observation, we detected SENP expression in hypertrophic mouse hearts using immunohistochemistry, and the same results were obtained (Figure 4B). RT‐qPCR revealed that the expression of SENP1 was increased by hypertrophic stress (Figure 4C). The results revealed increased mRNA and protein expression of SENP1 in hypertrophic NRCMs (Figure 4D,E).

We also validated the expression of SENP1 in human heart tissues and found that hypertrophic stimuli led to the abnormal induction of SENP1 at both the mRNA and protein levels in CMs (Figure S5, Supporting Information). We then constructed siSENP1–1 and siSENP1–2 and observed that the cell surface area of CMs was markedly increased in the siSENP1 group after Ang II stimulation (Figure S6, Supporting Information).

SUMOylation is reversed by six SENPs in mammalian cells, including SENP1–3 and SENP5–7.^[^ ^28^ ^]^ Notably, SENP1 and SENP2 can deconjugate all SUMO isoforms, whereas SENP3 and SENP5–7 preferentially deconjugate SUMO2/3‐modified proteins and SUMO chains.^[^ ^29^, ^30^ ^]^ Since TEAD1 is modified by SUMO1, we examined the potential role of SENP1–2 in TEAD1 deSUMOylation. To elucidate the deSUMOylation process of TEAD1, we transfected the plasmids FLAG‐SENP1, FLAG‐SENP2, MYC‐TEAD1, and HA‐SUMO1 into 293T cells. Through Co‐IP assays, we found that the overexpression of SENP1 attenuated the SUMOylation of TEAD1, whereas the overexpression of SENP2 only partially reduced this modification (Figure 4F). Consistent with this result, we performed siRNA targeting of SENP1 and SENP2 to determine their influence on the interaction between TEAD1 and SUMO1. The knockdown of SENP1, but not SENP2, increased the SUMOylation of TEAD1 in 293T cells (Figure 4G). Moreover, overexpression of wild‐type (WT) SENP1 blocked the SUMOylation of TEAD1, unlike overexpression of the catalytically inactive SENP1 mutant (Figure 4H). To provide further evidence, we assessed the relationship between TEAD1 and SENP1 in NRCM and AC16 cells during Ang II‐induced cardiac hypertrophy. The results demonstrated that Ang II stimulation increased the interaction between TEAD1 and SENP1 in a time‐dependent manner in both NRCM and AC16 cells (Figure 4I). Together, these findings suggest that the deSUMOylation of TEAD1 could be regulated by the deSUMOylation protease (deSUMOylase) SENP1.

### 2.4 K173 Is the Major SUMOylation Site in TEAD1

**Figure 5:**
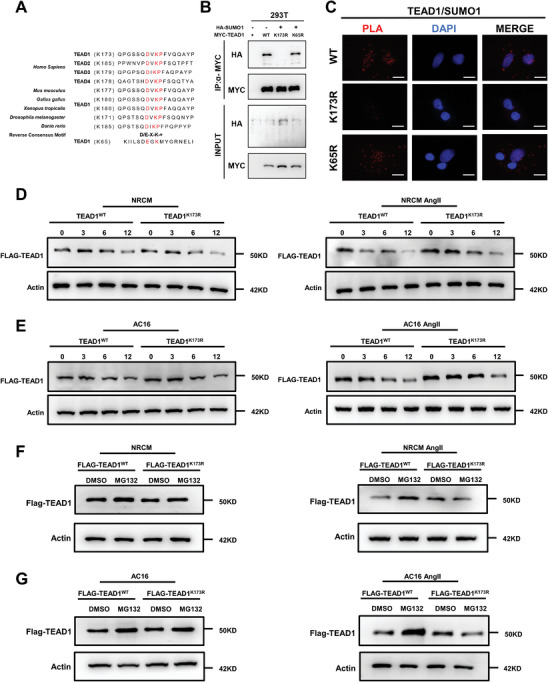
Lysine 173 (K173) is the major SUMOylation site in TEAD1. A) Sequenced and conserved SUMOylation modification sites in TEAD family members in different species. Potential SUMOylation sites are marked in red. B) HEK293T cells were transfected with HAP‐SUMO1 and MYC‐TEAD1 (WT, K65R, or K173R). MYC‐TEAD1 was immunoprecipitated followed by immunoblotting to detect SUMO1 (HA), and TEAD1 (MYC). C) Proximity ligation assay (PLA) of exogenous TEAD1 and SUMO1 in 293T cells co‐expressing MYC‐TEAD1‐WT, MYC‐TEAD1‐K65R, MYC‐TEAD1‐K173R, and HA‐SUMO1 plasmids. Scale bar, 10 µm. D–E) Immunoblotting analysis of FLAG‐TEAD1 in NRCMs and AC16 cells infected with FLAG‐TEAD1‐WT and FLAG‐TEAD1‐KR and treated with angiotensin II (1 µm) for 48 h and cycloheximide (50 µm) for the indicated durations (0, 3, 6, or 12 h). F,G) Immunoblotting analysis of FLAG‐TEAD1 in NRCMs and AC16 cells after FLAG‐TEAD1‐WT and FLAG‐TEAD1‐KR infection for 24 h, treatment with angiotensin II (1 µm) for 48 h, and dimethyl sulfoxide (DMSO) and MG132 (50 µm) treatment for 6 h.

To identify the potential TEAD1 SUMOylation site(s), we used the Group‐based Prediction System‐SUMO,^[^ ^31^, ^32^, ^33^ ^]^ Joint Analyzer of SUMOylation Site and SIMs,^[^ ^34^ ^]^ and SUMOplot analysis programs to predict the SUMOylation sites of TEAD1 (Table S2, Supporting Information). By overlapping the sites predicted by the algorithms, we identified two potential SUMOylation sites in TEAD1, lysine 65 (K65) and K173. Intriguingly, the K173R SUMOylation site was present in all four TEAD families and was conserved from *Danio rerio* to *Homo sapiens*. Both of the potential SUMOylation sites were in the canonical consensus SUMO motif φ‐K‐X‐D/E (**Figure** **5**A). To validate whether TEAD1 is SUMOylated at these two sites, we individually mutated K65 and K173 to the non‐SUMOylable residue arginine (R) and then transiently transfected WT, K65R, or K173R MYC‐TEAD1 mutants, together with HA‐SUMO1, into HEK293T cells. The results of the Co‐IP assay showed that the K173R mutation largely abolished the SUMOylation of TEAD1 (Figure 5B). We then performed PLA in 293T cells co‐expressing WT, K65R, and K173R MYC‐TEAD1 and HA‐SUMO1. Multiple red fluorescent puncta were visible in the nuclei of cells expressing MYC‐TEAD1‐WT and MYC‐TEAD1‐K65R, but not in those expressing MYC‐TEAD1‐K173R, indicating that K173 is the main SUMOylation site in TEAD1 (Figure 5C).

SUMOylation has important effects on cellular processes, including protein stability, the subcellular localization of proteins, and signal transduction.^[^ ^35^ ^]^ To investigate whether the stability of the TEAD1 protein is affected by SUMO modifications, we transfected Ad‐Flag‐TEAD1‐WT or Ad‐Flag‐TEAD1‐K173R into NRCM and AC16 cells, followed by the addition of cycloheximide to inhibit protein synthesis. In the absence of Ang II stimulation, the protein levels of both Ad‐Flag‐TEAD1‐WT and Ad‐Flag‐TEAD1‐KR slowly decreased with cycloheximide treatment, with no significant difference between them. We found that the rate of protein degradation in Ad‐Flag‐TEAD1‐WT was significantly higher than that in the Ad‐Flag‐TEAD1‐K173R mutant after Ang II stimulation, which was reversed by the peptide‐aldehyde proteasome inhibitor MG132 (Figure 5D–G). The results revealed that the increase in the protein stability of Ad‐Flag‐TEAD1‐KR was regulated mainly through inhibition of the ubiquitin‐proteasome pathway. These results suggest that SUMO modification reduces TEAD1 protein stability, whereas TEAD1 deSUMOylation enhances it.

### 2.5 TEAD1 SUMOylation Decreases the Interaction between TEAD1 and Its Co‐Activators YAP and TAZ

**Figure 6:**
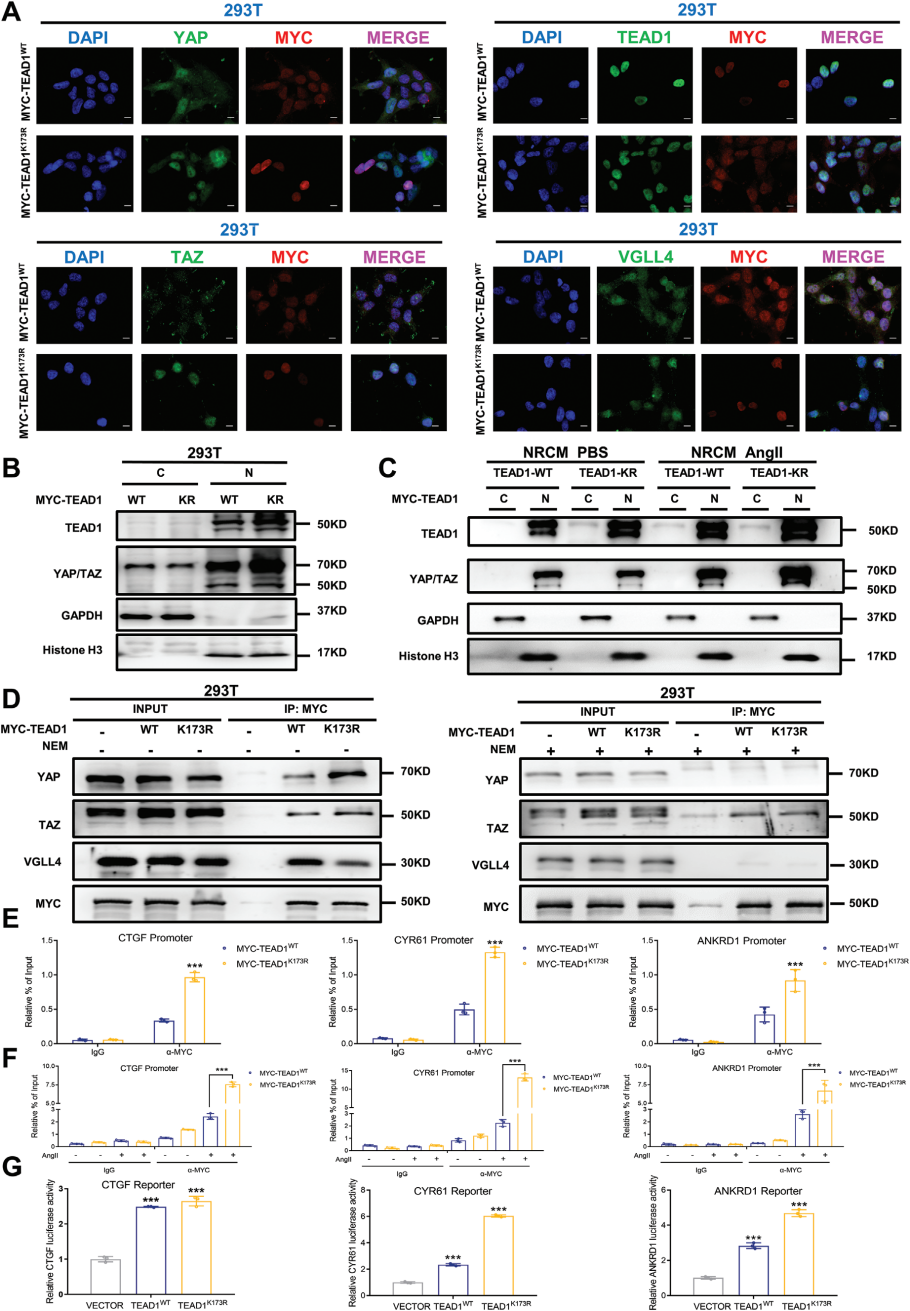
TEAD1 SUMOylation decreases the interaction between TEAD1 and its co‐activators YAP/TAZ. A) Localization of YAP, TAZ, TEAD1, and VGLL4 with MYC‐TEAD1 (red) observed by immunofluorescence staining after transfecting MYC‐TEAD1‐WT and MYC‐TEAD1‐K173R into 293T cells. The nuclei were stained with DAPI (blue). Scale bar, 10 µm. B) Representative western blots and quantitative results of TEAD1 and YAP/TAZ protein levels in the cytosolic and nuclear fractions after transfecting MYC‐TEAD1‐WT and MYC‐TEAD1‐K173R into 293T cells. C) Representative western blots and quantitative results of TEAD1 and YAP/TAZ protein levels in the cytosolic and nuclear fractions after transfecting MYC‐TEAD1‐WT and MYC‐TEAD1‐K173R into NRCMs with or without angiotensin II (1 µM) treatment for 48 h. D) HEK293T cells were transfected with the indicated plasmids. Cell lysates with or without NEM addition (20 mm) were subjected to immunoprecipitation with an anti‐MYC antibody and were then analyzed by western blotting. E,F) Promoters of CTGF, CYR61, and ANKRD1 in HEK293T cells transfected with MYC‐TEAD1‐WT or MYC‐TEAD1‐K173R plasmids, detected by chromatin immunoprecipitation (ChIP). *n* = 3 per group. G) Relative luciferase activity of HEK293T cells transfected with MYC‐TEAD1‐WT or MYC‐TEAD1‐K163R plasmids, and CTGF, CYR61, and ANKRD1 promoter luciferase reporter plasmids. *n* = 3 per group.

SUMOylation modulates gene transcription by regulating the recruitment of SUMOylated transcriptional co‐regulators. The transcriptional activity of TEAD1 is tightly regulated by the co‐activators YAP/TAZ and the co‐repressor VGLL4. Thus, our study aimed to elucidate the potential influence of TEAD1 SUMOylation on the interplay between YAP/TAZ and VGLL4. To further investigate the effects of SUMO modification on subcellular localization, we performed immunofluorescence staining of 293T cells transfected with MYC‐TEAD1‐WT and MYC‐TEAD1‐K173R. We found that the nuclear localization of YAP/TAZ was considerably affected. The results revealed that K173R induced high YAP/TAZ expression in the nucleus, thus activating YAP/TAZ. However, both TEAD1‐WT and the TEAD1‐K173R mutant showed similar TEAD1 nuclear staining patterns. Meanwhile, no significant change in the nucleocytoplasmic localization of VGLL4 was observed between the TEAD1‐WT and TEAD1‐K173R mutant (**Figure** **6**A). Subsequently, we proceeded to corroborate the augmented nuclear expression of YAP/TAZ mediated by K173R via western blotting of nuclear and cytosolic proteins in both the 293T cell line and NRCMs (Figure 6B,C). We initially conducted IP assays in 293T cells, revealing that the TEAD1‐K173R mutation enhanced the association between YAP/TAZ and TEAD1 while concurrently reducing the binding affinity of VGLL4 and TEAD1 (Figure 6D). NEM, a derivative of maleic acid, acts as an irreversible inhibitor of cysteine proteases via the alkylation of free sulfhydryl groups.^[^ ^36^ ^]^ Consequently, NEM is widely employed as an SENP inhibitor in lysis buffers to inhibit protein deSUMOylation during cellular lysis and IP. Interestingly, inclusion of NEM in the lysis buffer disrupted the interaction between TEAD1 and YAP/TAZ. Similarly, we observed a comparable outcome regarding the interaction of VGLL4/TEAD1; the addition of NEM to the lysis buffer significantly impaired this interaction (Figure 6D). These results highlight the disruptive impact of NEM treatment on the TEAD1, YAP/TAZ, and TEAD1/VGLL4 protein complexes, suggesting its potential role as an inhibitor. NEM in the lysis buffer is not a feasible approach to assess SUMO‐mediated protein–protein interactions between TEAD1 and its binding partners. Interestingly, chromatin IP (ChIP) assays showed that the classical YAP/TAZ downstream target genes CTGF, CYR61, and ANKRD1 were dramatically affected by MYC‐TEAD1‐WT and K173R mutants. The K173R mutation of TEAD1 enhanced the transcriptional activation of CTGF, CYR61, and ANKRD1 reporters in 293T cells compared to the TEAD1‐WT (Figure 6E). To confirm these results, Ang II stimulation was performed in AC16 cells, and we found that K173 significantly augmented the transcriptional activation of the CTGF, CYR61, and ANKRD1 reporters compared to the WT (Figure 6F). Luciferase reporter assay showed that the TEAD1‐K173R mutant significantly increased the luciferase activity of the CTGF, CYR61, and ANKRD1 luciferase reporters (Figure 6G). These findings suggest that the SUMOylation of TEAD1 may play a regulatory role in its function through the Hippo signaling pathway, thereby influencing its subcellular localization and association with chromatin.

### 2.6 TEAD1 deSUMOylation Aggravates TAC‐Induced Cardiac Hypertrophy in Mice

**Figure 7:**
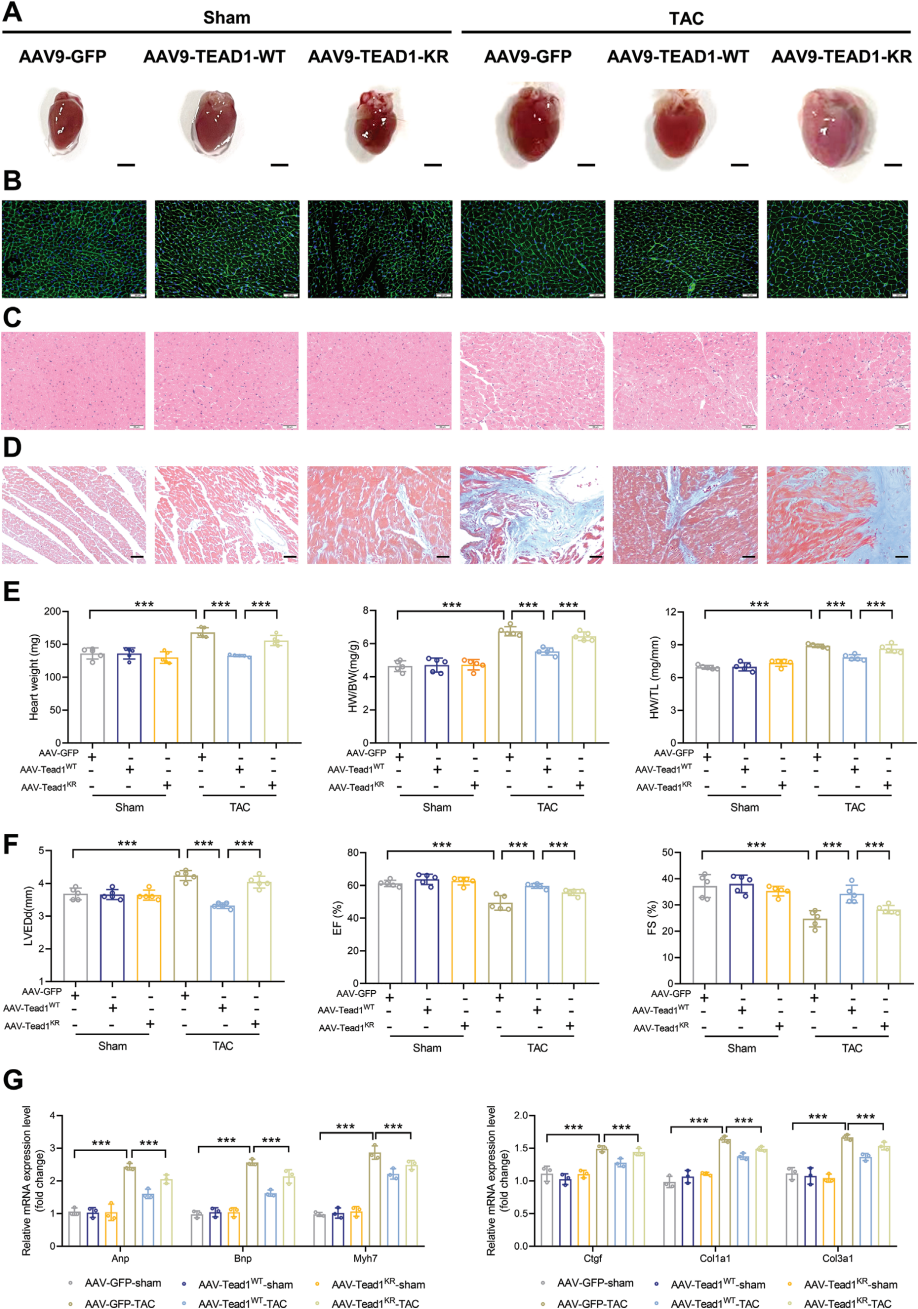
TEAD1 deSUMOylation aggravates TAC‐induced cardiac hypertrophy in mice. A) Representative images of the gross appearance of heart in AAV‐GFP, AAV‐TEAD1‐WT, and AAV‐TEAD1‐K177R groups at 4 weeks after sham or TAC surgery. n = 5 mice per group. Scale bar, 1 mm. B–D) Representative images of hematoxylin–eosin (HE) staining (B), cell boundaries demarcated with fluorescein isothiocyanate‐wheat germ agglutinin (WGA) (C), and Masson staining (D) of the hearts in the AAV‐GFP, AAV‐TEAD1‐WT, and AAV‐TEAD1‐K177R groups 4 weeks after sham or TAC surgery. n = 5 mice per group. Scale bar, 20 µm. E) Heart weight (HW), HW/body weight (BW), lung weight (LW)/BW, and HW/tibia length (TL) ratios in the AAV‐GFP, AAV‐TEAD1‐WT, and AAV‐TEAD1‐K177R groups 4 weeks after sham or TAC surgery. n = 10 mice per group. F) Assessments of echocardiographic parameters of left ventricular (LV) end‐diastolic dimension (LVEDd), LV end‐systolic dimension (LVESd), ejection fraction (EF), and fraction shortening (FS) in the AAV‐GFP, AAV‐TEAD1‐WT, and AAV‐TEAD1‐K177R groups 4 weeks after sham or TAC surgery. *n* = 10 mice per group. G) Representative RT‐qPCR and quantitative results for hypertrophy and fibrosis marker genes in heart tissues from the indicated mice. *n* = 3 mice per group.

To evaluate the function of TEAD SUMOylation in pathological myocardial hypertrophy in vivo, we pre‐injected mice with CM‐specific adeno‐associated virus (AAV) to deliver TEAD1‐WT and TEAD1‐K177R. Since the mutant of K173 in humans but K177 in mice, to clarify the function of TEAD1 in vivo, we measured TEAD1 expression in Ad‐GFP, Ad‐Flag‐TEAD1‐WT, and Ad‐Flag‐TEAD1‐K177R mice. We found no difference in TEAD1 expression between Ad‐Flag‐TEAD1‐WT and Ad‐Flag‐TEAD1‐K177R mice (Figure S7, Supporting Information). Four weeks after TAC, AAV‐TEAD1‐K177R mice showed larger heart sizes and larger CM cross‐sectional areas in the TAC‐induced cardiac hypertrophy model (**Figure** **7**A–C). Furthermore, AAV‐TEAD1‐K177R mice exhibited exacerbated TAC‐induced cardiac fibrosis, as shown by Masson's staining of heart sections (Figure 7D). We found that the heart weight/body weight, lung weight/body weight, and heart/tibia length ratios in AAV‐TEAD1‐K177R mice were substantially higher than those in AAV‐TEAD1‐WT mice (Figure 7E). In addition, echocardiographic evaluation of left ventricular function and architecture, including left ventricular end‐diastolic diameter, fractional shortening, and ejection fraction, further verified the worsened cardiomegaly and reduced myocardial function in AAV‐TEAD1‐K177R mice compared to AAV‐TEAD1‐WT mice (Figure 7F). The mRNA levels of genes related to cardiac hypertrophy (Anp, Bnp, and Myh7) and fibrosis (Col1a1, Col3a1, and Ctgf) were upregulated in AAV‐TEAD1‐K177R mice (Figure 7G). The protein expression levels of these genes were also higher in AAV‐TEAD1‐K177R mice than in AAV‐TEAD1‐WT mice (Figure S8, Supporting Information). Overall, AAV‐TEAD1‐WT effectively inhibited TAC surgery‐induced cardiac hypertrophy, heart dysfunction, and heart remodeling whereas AAV‐TEAD1‐K177R aggravated pressure overload‐induced cardiac hypertrophy.

### 2.7 TEAD1 SUMOylation Regulates Oxidative Stress in CM Hypertrophy

**Figure 8:**
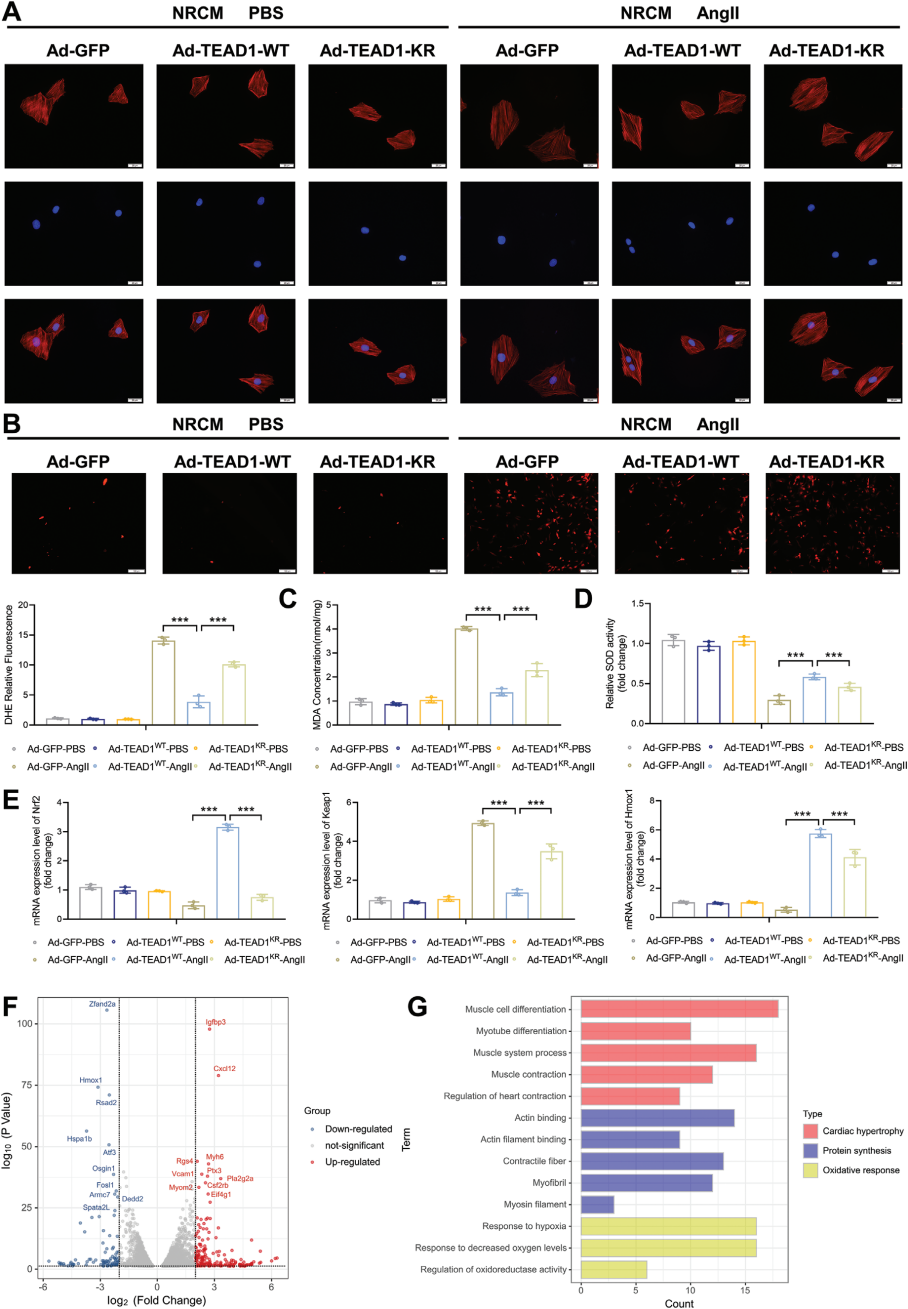
TEAD1 SUMOylation regulates oxidative stress in cardiomyocyte hypertrophy. A) Representative immunofluorescence images of F‐actin (red) staining of NRCMs infected with Ad‐GFP, Ad‐TEAD1‐WT, and Ad‐TEAD1‐KR with or without angiotensin II treatment (1 µm). The nuclei were stained with DAPI (blue). Scale bar, 20 µm. B) Dihydroethidium (DHE) staining (above) and quantitative results of DHE relative fluorescence (below) in NRCMs infected with the indicated adenovirus and incubated with or without angiotensin II (1 µm) for 48 h. n = 20 fields. Scale bar, 100 µm. C) Malondialdehyde (MDA) content of NRCMs infected with the indicated adenovirus and incubated with or without angiotensin II (1 µm) for 48 h. n = 3 mice per group. D) Relative superoxide dismutase (SOD) activity of NRCMs infected with the indicated adenovirus and incubated with or without angiotensin II (1 µm) for 48 h. n = 3 mice per group. E) Representative RT‐qPCR and quantitative results of Nrf2, Keap1, and Hmox1 in NRCMs after infection with the indicated adenovirus and incubation with or without angiotensin II (1 µm) for 48 h. n = 3 mice per group. F) Volcano plot of differentially expressed genes (DEGs) with a cutoff of |log2 (fold change) | > 2 and *p* value < 0.05. G) Gene ontology analysis of molecular events involved in cardiac hypertrophy, protein synthesis, and oxidative response in RNA‐sequencing data.

To further clarify the role of TEAD1 SUMOylation in CM enlargement, we constructed adenovirus‐expressing TEAD1‐WT (Ad‐TEAD1‐WT) and TEAD1‐K173R (Ad‐TEAD1‐K173R) to infect NRCMs (Figure S9, Supporting Information). The cell surface area of CMs was markedly increased in the Ad‐TEAD1‐K173R cell group, whereas Ad‐TEAD1‐WT notably inhibited this increase (**Figure** **8**A). DeSUMOylation of TEAD1 abrogated the effect of TEAD1 on antioxidant stress in hypertrophic CMs as shown by dihydroethidium staining (Figure 8B). Furthermore, the malondialdehyde (MDA) content was reduced by TEAD1 overexpression but superoxide dismutase (SOD) activity was enhanced; TEAD1‐K173R showed the opposite result (Figure 8C,D). Peroxiredoxin 1 significantly upregulated the expression of NRF2, Kelch‐like ECH‐associated protein 1, and heme oxygenase‐1 (HO1), which are recognized anti‐oxidative stress factors in CMs (Figure 8E). To determine the impact of TEAD1 SUMOylation at the molecular level, we performed RNA sequencing analysis on NRCMs infected with Ad‐TEAD1‐WT and Ad‐TEAD1‐K173R with or without Ang II stimulation. Differentially expressed genes (DEGs) were identified using a cutoff of |log2 (fold change) | > 2 and *p* < 0.05. We identified 181 upregulated and 96 downregulated genes (Figure 8F). Gene set enrichment analysis revealed that TEAD1‐WT and TEAD1‐K173R‐regulated DEGs were enriched for cardiac hypertrophy, protein synthesis, and oxidative response (Figure 8G). Overall, these data indicate that the SUMOylation of TEAD1 regulates CM enlargement and oxidative stress during cardiac hypertrophy.

## 3 Discussion

Pathological cardiac hypertrophy is one of the most important components of the pathogenesis of heart failure.^[^ ^37^ ^]^ Unlike physiological hypertrophy, the triggers and signaling mechanisms that cause this maladaptive response include fibrosis, apoptosis, and various cellular and structural dysfunctions.^[^ ^38^ ^]^ In recent years, significant progress has been made in the identification of key molecular targets and signaling pathways involved in pathological cardiac hypertrophy.^[^ ^2^, ^4^, ^39^ ^]^ Despite this progress, current clinical pharmacological interventions for effectively treating cardiac hypertrophy remain unsatisfactory.

The TEAD transcription factor family, comprising transcriptional enhancer factors, includes four members (TEAD1–4), that are broadly expressed in embryonic and adult tissues.^[^ ^40^, ^41^, ^42^ ^]^ In this study, we demonstrated that the protein and mRNA expression patterns of TEAD1 were significantly upregulated in the heart tissues of patients diagnosed with hypertrophic cardiomyopathy. We observed that TEAD1 expression was dramatically upregulated in cardiac hypertrophy induced by pressure overload in vivo and Ang II in vitro. Consistently, compared to negative controls, the cell surface area of CMs in the siTEAD1 group was markedly increased, and biomarkers of cardiac hypertrophy were significantly upregulated. These results suggest that TEAD1 exerts cardioprotective effects against cardiac hypertrophy and may be an effective therapeutic target for pathological cardiac hypertrophy.

Compared to classical post‐translational modifications, such as phosphorylation, ubiquitination, and glycosylation, SUMOylation is a relatively new and unique post‐translational modification characterized by highly dynamic and reversible processes.^[^ ^18^, ^43^, ^44^ ^]^ SUMOylation has been extensively studied for its ability to modulate various cellular processes. Emerging evidence shows that SUMOylation is an important reversible post‐translational modification that contributes to the maintenance of cardiac homeostasis in response to hypertrophic stimuli.^[^ ^45^, ^46^ ^]^ Recent studies have shown that SENP1 protects against cardiac remodeling and dysfunction triggered by long‐term pressure overload. SENP1 was initially thought to protect against myocardial ischemia/reperfusion injury. SENP1 deficiency weakens cardiac function and accelerates CM apoptosis, and these roles are regulated by the hypoxia‐inducible factor 1α‐dependent pathway.^[^ ^27^ ^]^ Furthermore, animal models lacking SENP1 exhibit exacerbated cardiac hypertrophy under stress stimuli, indicating a protective role for SENP1 in pathological cardiac remodeling. Conversely, overexpression of SENP1 attenuates hypertrophic growth and improves cardiac function. Our data revealed that the post‐translational SUMOylation of TEAD1 in response to cardiac hypertrophy stimulation was mediated by the deSUMOylase of SENP1. Here, we clearly demonstrate that the SENP1‐regulated SUMOylation of TEAD1 functions as an intrinsic regulatory mechanism for TEAD1 activation and on/off Hippo signaling, influencing protein stability, subcellular localization, and chromatin association. The in vitro results were confirmed in vivo. Our study provides the first evidence that the SUMOylation of TEAD1 can significantly modulate TAC‐induced cardiac hypertrophy. Therefore, targeting the SUMOylation of TEAD1 may represent a promising strategy for treating pathological cardiac hypertrophy.

Based on global gene expression profiling using RNA sequencing, we systematically revealed that the DEGs between TEAD1‐WT and TEAD1‐K173R in NRCMs were related to oxidative stress, cardiac hypertrophy, and protein synthesis. It is well known that oxidative stress plays an important role in cardiac hypertrophy.^[^ ^47^, ^48^, ^49^ ^]^ As an important transcription factor, NRF2 controls cellular defense responses against oxidative stress by balancing redox signaling and protecting against oxidative insults. NRF2 and its downstream target HO1 protect against myocardial hypertrophy, myocardial ischemia‐reperfusion, and other myocardial injuries.^[^ ^49^, ^50^, ^51^ ^]^ As a stress‐inducible enzyme, HO1 catalyzes heme degradation to release free iron, carbon monoxide, and biliverdin from mammalian cells.^[^ ^52^ ^]^ Overall, these data indicate that the SUMOylation of TEAD1 regulates CM enlargement and oxidative stress during cardiac hypertrophy. These findings provide new insight into the pathogenesis of myocardial hypertrophy and suggest novel therapeutic strategies for this disease.

## 4 Conclusion

In this study, we provide significant evidence of a strong association between TEAD1 and pathological cardiac hypertrophy. We observed that, during cardiac hypertrophy, the modification of TEAD1 by SUMO1 was regulated by the SUMO protease SENP1. K173 is a crucial site for SUMO‐mediated TEAD1 modification. Mutations at this site not only impact the protein stability of TEAD1, but also lead to enhanced interaction between TEAD1 and its transcriptional co‐activator YAP, affecting the localization of TEAD1 in the cytosol and nucleus and the transcription of downstream target genes. In animal models, AAV‐TEAD1‐WT effectively inhibited TAC surgery‐induced cardiac hypertrophy, heart dysfunction, and heart remodeling, whereas AAV‐TEAD1‐K177R aggravated pressure overload‐induced cardiac hypertrophy. Mechanistically, Nrf2‐Hmox1 is a potential target of the oxidative stress in cardiac hypertrophy induced by the SUMOylation of TEAD1. Overall, these findings offer a novel perspective on the mechanisms underlying pathological cardiac hypertrophy and present innovative therapeutic strategies for this disease.

## 5 Experimental Section

### Human Tissue Specimens

Two types of human heart tissues were collected for this study: normal donors (n = 5) and patients with heart failure (n = 5). The samples were obtained from the left ventricular apex. The normal control samples were obtained from healthy donors who died of noncardiac causes. The heart failure samples were collected from patients who underwent heart transplantation. Written informed consent was obtained for all donors and transplant patients. All procedures involving human heart tissue were performed in accordance with the principles of the Declaration of Helsinki. This study was approved by the Human Research Ethics Committee of the Shanghai Chest Hospital, Shanghai, China (Permit No. KS(Y)21240).

### Animal Models

Animals were maintained at the Center for Experimental Animals, Shanghai Chest Hospital, Shanghai, China. Male mice aged 8–10 weeks (body weight 23–25 g) were used. The animals were housed under a 12 h light‐dark cycle and received water and food ad libitum using a standard chow diet. For the AAV‐infected mice, male 6–8‐week‐old C57BL/6J mice were randomly chosen to receive a single‐bolus tail vein injection (100 µL per mouse) of either AAV9 encoding TEAD1‐WT and TEAD1‐K177R or AAV9 encoding pcAAV2/9‐CMV‐EGFP‐P2A‐WPRE at 1 × 10^12^ vg mL^−1^. After two weeks, the animal model was established by TAC‐induced pressure overload, as previously described.^[^ ^53^ ^]^ Briefly, male mice (body weight 23–25 g) were anesthetized with pentobarbital sodium (30 mg kg^−1^; Sigma‐Aldrich, catalog no. P3761) via intraperitoneal injection. The left chest was opened to expose the thoracic aorta. The exposed aorta was constricted using 6‐0 silk sutures, which were tied against a 27‐gauge needle, followed by needle removal and thoracic cavity closure. Sham‐operated mice underwent a similar procedure without ligation. All animal experiments and procedures were conducted in compliance with the ethical regulations for animal testing and welfare. All procedures involving experimental mice and rats were performed according to protocols approved by the Committee for Animal Research of Shanghai Chest Hospital and conformed to the Guidelines for the Care and Use of Laboratory Animals (Permit No. KS23042).

### Echocardiography

Echocardiography was performed to evaluate the cardiac function at specified time points, as previously described. A small animal ultrasound imaging system (Vevo 2100, Fujifilm, VisualSonics, Canada), equipped with a 30 MHz linear ultrasonic transducer, was used.

### Plasmids and siRNA

Plasmids encoding full‐length TEAD1, SUMO1, SUMO2, SUMO3 UBC9 SENP1, SENP2 were cloned from cDNAs and ligated to pcDNA3 vector. TEAD1 KR mutants, and SENP1/2 CS mutants were constructed based on this vector. SENP1, SENP2 siRNA and rat TEAD1 siRNA and the scramble control were purchased from Gene Pharma, and the siRNA sequences were as follows:

SENP1 siRNA‐1: sense 5′‐GCGGGAACATTCAGTACATGA‐3′

SENP1 siRNA‐2: sense 5′‐ TACTGGAACTAAGACATCGA‐3′

SENP2 siRNA‐1: sense 5′‐ GCAAAGGTAATCCAGAGAGTT‐3′

SENP2 siRNA‐2: sense 5′‐ GGAGCCTGACCTATCAGAA −3′

RAT‐TEAD1 siRNA‐1: sense 5′‐CACAAGACGTCAAGCCCTTTGTGCA‐3′;

RAT‐TEAD1 siRNA‐2: sense 5′‐AGACGGAGTATGCGAGGTT‐3′.

### Cell Culture and Transfection

NRCMs were isolated from 24‐h‐old Sprague–Dawley rats obtained from SLAC Laboratory Animal Ltd. (Shanghai, China). Briefly, the hearts were cut into pieces, washed in ice‐cold phosphate‐buffered saline (PBS), and predigested for 3 min with 0.125% trypsin at 37 °C, followed by 90 min digestion with 1 mg mL^−1^ collagenase A (Roche) at 37 °C under constant shaking (60 rpm). The digested cell suspension was filtered through a 70 µm cell strainer and then plated onto cultured dishes. NRCMs were separated from fibroblasts by pre‐plating the digested cell suspension for 50 min. NRCMs were maintained at 37 °C with 5% CO_2_ in Dulbecco's modified Eagle's medium/low glucose supplemented with 10% fetal bovine serum.

CMs and non‐CMs were isolated from adult mouse hearts using a simplified method as previously described.^[^ ^5^, ^54^ ^]^ Briefly, the hearts of C57/BL6J male mice, aged 8–12 weeks, were perfused with 10 mL EDTA buffer to stop their beating. Digestion was achieved using 10 mL of EDTA buffer, 3 mL of perfusion buffer, and 30–50 mL of collagenase buffer. The constituent chambers, including the atria, left ventricle, and right ventricle, were gently separated into 1 mm pieces. Cellular dissociation was completed by gentle trituration, and enzyme activity was inhibited by the addition of 5 mL stop buffer. CMs were separated into cell pellets by four sequential rounds of gravity settling, and the supernatant from each round was combined to produce a fraction containing non‐CM cardiac populations.

H9C2, AC16, and HEK293T cells were obtained from the American Type Culture Collection and cultured in Dulbecco's modified Eagle's medium/high‐glucose supplemented with 10% fetal bovine serum, 100 units mL^−1^ penicillin, and 100 µg mL^−1^ streptomycin (Yeasen Biotech) at 37 °C in 5% CO_2_. Transient transfection was performed using polyethylenimine (Polysciences) or Lipofectamine 2000 (Invitrogen), according to the manufacturer's instructions. NRCMs and AC16 cells stably expressing FLAG‐TEAD1‐WT and FLAG‐TEAD1‐K173R were generated by transduction of the indicated lentivirus vectors, and the stable cells were selected for at least one week using puromycin (1 µg mL^−1^). Stable cell pools containing a mixture of clones were used for further analysis.

### Western Blotting and Co‐IP

Western blotting and Co‐IP were performed as described previously.^[^ ^55^, ^56^ ^]^ For the endogenous SUMOylation assay, cells were harvested in ice‐cold PBS and lysed in lysis buffer (150 mM NaCl, 50 mM Tris–HCl[pH 7.4], 1 mM EDTA, 0.3% NP‐40, and complete protease inhibitor) containing 20 mM NEM (Sigma). The cell lysates were mixed with TEAD1 (Abcam, ab221367), SUMO1 (CST, 4930), or SUMO2/3 (CST, 4971) antibodies, pre‐incubated with protein A/G magnetic beads (Thermo Scientific) for 2 h, and washed three times with cold lysis buffer. For the exogenous SUMOylation assay, exogenous HA‐SUMO1 or HA –SUMO2/3 were co‐transfected with MYC‐FLAG‐TEAD1 or MYC‐FLAG‐TEAD1‐K173R in HEK293T cells. After 48 h, the cells were harvested with SUMO lysis buffer and then directly incubated with HA‐beads (Thermo Pierce, 88836) or FLAG beads (Sigma, M8823). Co‐precipitates with primary antibodies were separated by SDS‐PAGE.

### Immunohistochemistry and Immunofluorescence

Immunohistochemical staining was performed as previously described.^[^ ^57^ ^]^ Briefly, tissues were fixed in 4% paraformaldehyde and embedded in paraffin, in accordance with standard procedures. After antigen retrieval in pH 8.0 sodium citrate solution, the heart sections were blocked for peroxidases and non‐specific binding of antibodies using 3% H_2_O_2_‐methanol solution and 5% bovine serum albumin (BSA) solution, respectively, for 20 min each and then washed with PBS. Subsequently, the sections were incubated overnight with TEAD1 (Abcam, ab221367), TEAD2 (Proteintech, 21159‐1‐AP) TEAD3 (Proteintech, 13120‐1‐AP), TEAD4 (Santa Cruz, sc‐390578) and SENP1 (Abcam, ab236094) antibodies at 4 °C. After washing with PBS three times, the sections were incubated with secondary antibody, either goat anti‐mouse or rabbit IgG conjugated with peroxidase, at room temperature for 60 min, counterstained with hematoxylin, and sealed with neutral resin. Images were captured using an Olympus IX73 microscope.

For immunofluorescence staining of the heart tissues, after similar treatment with the primary antibody, the samples were incubated for 1 h at room temperature with the corresponding fluorescent secondary antibodies conjugated to Alexa Fluor 488 or 594. 4′,6‐Diamidino‐2‐phenylindole (DAPI) was used for nuclear staining. The images were captured using a fluorescence microscope (IX73, Olympus). The following primary antibodies were used: anti‐troponin T‐C (Proteintech, 15513‐1‐AP), anti‐TEAD1 (Santa Cruz, sc‐393976), rabbit IgG (Santa Cruz Biotechnology, sc‐2026) and mouse IgG (Santa Cruz Biotechnology, sc‐2025). Alexa Fluor 488 goat anti‐rabbit IgG (Beyotime Biotech, A0423) and CY3 goat anti‐mouse IgG (Beyotime Biotech, A0521) were used as secondary antibodies.

For immunofluorescence staining, cells were placed on coverslips (Thermo Fisher Scientific) overnight before treatment. After treatment with or without 1 µM Ang II in serum‐free medium for 24 h, the cells were rinsed in ice‐cold PBS, fixed with 4% (v/v) paraformaldehyde at room temperature for 30 min, and permeabilized in 0.3% Triton X‐100 for 10 min. Cells were then blocked for 30 min at room temperature with 3% (w/v) BSA and then incubated with primary antibodies in 1% BSA at 4°C overnight. They were then rinsed three times in PBS and incubated with the appropriate secondary antibodies conjugated with Alexa‐594 or Alexa‐488 for 1 h. The cells were then washed three times with PBS and sealed with DAPI (Thermo Fisher Scientific, P36966). Images were obtained using an Olympus IX73 microscope.

Alexa Fluor 594 Phalloidin (Invitrogen, A12381) staining was used to evaluate CM size. Cells were fixed with 4% paraformaldehyde, permeabilized with 0.1% Triton X‐100, and blocked with 10% BSA, followed by incubation with Alexa Fluor 594 at 37°C for 1 h. Images were obtained using an Olympus IX73 microscope; in each group, more than 20 CMs were measured using Image J software (National Institutes of Health).

### RNA Sequencing, qPCR, and ChIP‐qPCR

Total RNA from NRCMs stably expressing Ad‐GFP, Ad‐Flag‐TEAD1‐WT, or Ad‐Flag‐TEAD1‐K173R was extracted and used for RNA‐seq on the HiSeq 2500 platform. DEGs were analyzed using DESeq2 software and verified by qPCR. Total RNA was isolated from the heart tissues of patients, NRCMs, AC16 cells, and H9C2 cells using TRIzol reagent (Invitrogen, 15596026), following the manufacturer's instructions. RNA was reverse transcribed using the HiScript III 1st Strand cDNA Synthesis Kit (Vazyme, R312) according to the manufacturer's protocol. Real‐time polymerase chain reaction was performed using AceQ Universal SYBR qPCR Master Mix (Vazyme, Q511) according to the manufacturer's instructions. The mRNA expression levels of the target genes were normalized to the 18S rRNA expression levels. ChIP assays were performed using a chromatin IP kit (Millipore, 17–371) according to the manufacturer's instructions. FLAG‐bound chromatin fragments were immunoprecipitated from NRCMs stably expressing pLVX, pLVX‐FLAG‐TEAD1‐WT, or pLVX‐FLAG‐TEAD1‐K173R, followed by elution with ChIP elution buffer. The primers used are listed in Table S5 (Supporting Information).

### Luciferase Reporter Assay

Cells were transfected with Renilla luciferase plasmid and then co‐transfected with the CTGF, or CYR61, or ANKRD1 promoter luciferase reporter plasmid together with expression plasmid TEAD1WT or TEAD1K173R plasmids as indicated in figures. After 48 h, the cells were lysed using a passive lysis buffer and luciferase activity was measured using a dual‐luciferase reporter assay (Promega, E1910) and normalized to the activity of Renilla luciferase.

### Proximity Ligation Assay

Tissue PLA was performed using the reagents provided in the Duolink II in situ Brightfield kit (Sigma, DUO92012) according to the manufacturer's instructions. After standard formalin‐fixed, paraffin‐embedded section treatment and additional blocking with endogenous peroxidase (5 min at room temperature) and non‐specific protein binding (1 h at 37 °C), sections were incubated overnight at 4 °C with primary antibody. To detect canonical SUMOylation complexes in TEAD1, SUMO1, and SUMO2/3, mouse PLUS and rabbit MINUS secondary PLA antibodies were prepared according to the manufacturer's instructions. Ligation and amplification reactions were performed on all slides, as described by the manufacturer. Single‐stranded rolling circle amplification probes were visualized by reaction with a horseradish peroxidase‐labeled hybridization probe, again following the manufacturer's instructions. After washing twice in water, the sections were counterstained with hematoxylin (Sangon Laboratories) and sealed with neutral resin. For PLA, the coverslips were fixed in 4% paraformaldehyde for 30 min at room temperature. This was followed by standard immunofluorescence staining before secondary antibody incubation. For exogenous PLA, anti‐HA and anti‐FLAG antibodies were used. For endogenous PLA, TEAD1 was paired with SUMO1, SUMO2/3, or SENP1, and the PLA protocol was conducted. Briefly, two PLA probes (PLUS and MINUS commercial stocks) were mixed and diluted at a ratio of 1:5 with the antibody diluent buffer provided with the PLA probe kit. Enough of this mix was prepared to cover the sample and prevent drying during incubation. Subsequently, slides were incubated using ligation and amplification procedures. Cells were then sealed using ProLong Diamond Antifade Mountant with DAPI. Images were obtained using an Olympus IX73 microscope. The PLA puncta in each cell were counted for statistical analysis. The number of interactions (puncta per nucleus) calculated using the algorithm was recorded as the mean (± standard error) of ten independent fields of view.

### Lipid Peroxidation MDA Assay

A lipid peroxidation MDA assay kit (S0131S, Beyotime) was used to detect the MDA content. NRCMs were lysed in 150 mM IP buffer for 30 min at 4 °C. A standard sample was diluted to 1, 2, 5, 10, 20, and 50 µM with ddH_2_O for the production of a standard curve. Subsequently, the standard and test samples were incubated with MDA working solution at 100 °C for 15 min, following the manufacturer's instructions. The absorbance of the samples was measured at 532 nm using a SpectraMax i3X Multi‐Mode Microplate Reader.

### SOD Assay

For the SOD assay, NRCMs were lysed using the SOD sample preparation solution in the Total Superoxide Dismutase Assay Kit (Beyotime, S0101S). The SOD content was quantified by measuring the absorbance at 450 nm using a SpectraMax i3X Multi‐Mode Microplate Reader (Molecular Devices), following the manufacturer's instructions.

### Statistical Analysis

Statistical analysis was performed using GraphPad Prism 6 and SPSS 22.0. The significant differences between different data were evaluated by unpaired two‐tailed *t*‐test (for two‐sample comparisons) and one‐way ANOVA or two‐way ANOVA (for comparisons between multiple groups (≥3 groups)) followed by the Tukey's and Dunnett's multiple comparisons test. All data are expressed as the mean ± standard deviation (SD). P values less than 0.05 were considered significant (^*^ *p* < 0.05; ^**^ *p* < 0.01; ^***^ *p* < 0.001).

## Conflict of Interest

The authors declare no conflict of interest.

## Author Contributions

X.S., X.D., Z.H., and Y. L. contributed equally to this work. B.H., C.P., X.S., and Z.H. designed the study. X.D. Y.L. Z.J., and F.L. performed the experiments. H.T., F.Z., Y.L., and Z.C. analyzed the data. X.S., C.G., and X.W. drafted the manuscript. All authors read and approved the final manuscript.

## Supporting information

Supporting Information

Supporting Information

Supporting Information

## Data Availability

The data that support the findings of this study are available on request from the corresponding author. The data are not publicly available due to privacy or ethical restrictions.
