# Supplementary material for: SUMOylation of TEAD1 Modulates the Mechanism of Pathological Cardiac Hypertrophy
Source: Adv Sci (Weinh). 2024 Jan 15;11(12):2305677. doi: 10.1002/advs.202305677 (PMC10966521; doi:10.1002/advs.202305677)
Supplement: Supplementary file 1 — Supporting Information [file ADVS-11-2305677-s001.pdf]

ADVANCED SCIENCE Open Access

Supporting Information

for *Adv. Sci.*, DOI 10.1002/advs.202305677

SUMOylation of TEAD1 Modulates the Mechanism of Pathological Cardiac Hypertrophy

Xin Shi, Xuening Dang, Zhenyu Huang, Yanqiao Lu, Huan Tong, Feng Liang, Fei Zhuang, Yi Li, Zhaohua Cai, Huanhuan Huo, Zhaolei Jiang, Changqing Pan, Xia Wang, Chang Gu* and Ben He**

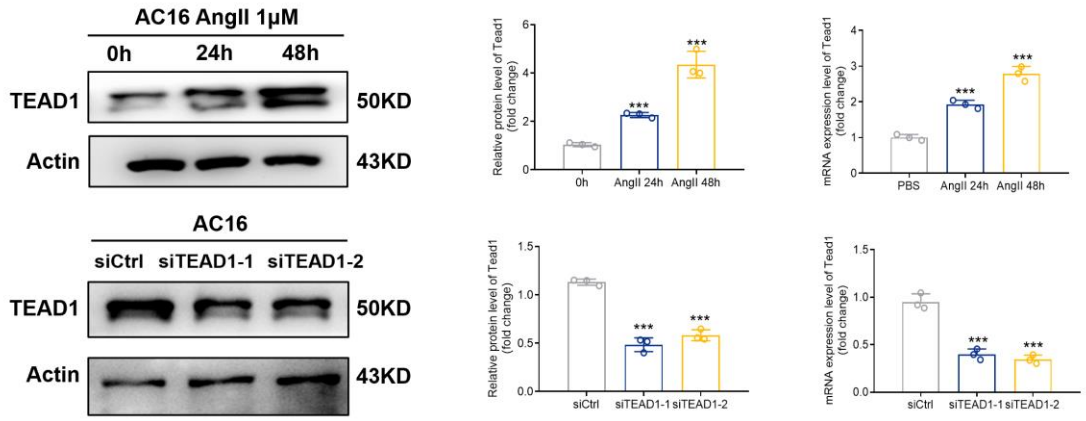

Figure S1. (A)Representative western blots and quantitative results of TEAD1 in AC16 cells stimulated with or without angiotensin II (1μM) for 24h or 48 h. n = 3 samples per group. (B) Representative RT-qPCR and quantitative results of TEAD1 mRNA levels in AC16 cells stimulated with or without angiotensin II (1μM) for 24h or 48 h. n = 3 samples per group. (C) Representative western blots and quantitative results of TEAD1 in AC16 cells transduced with siTEAD1-1, siTEAD1-2 or siControl. n = 3 samples per group. (D) Relative mRNA levels of TEAD1 in AC16 cells transduced with siTEAD1-1, siTEAD1-2 or siControl. n = 3 samples per group.

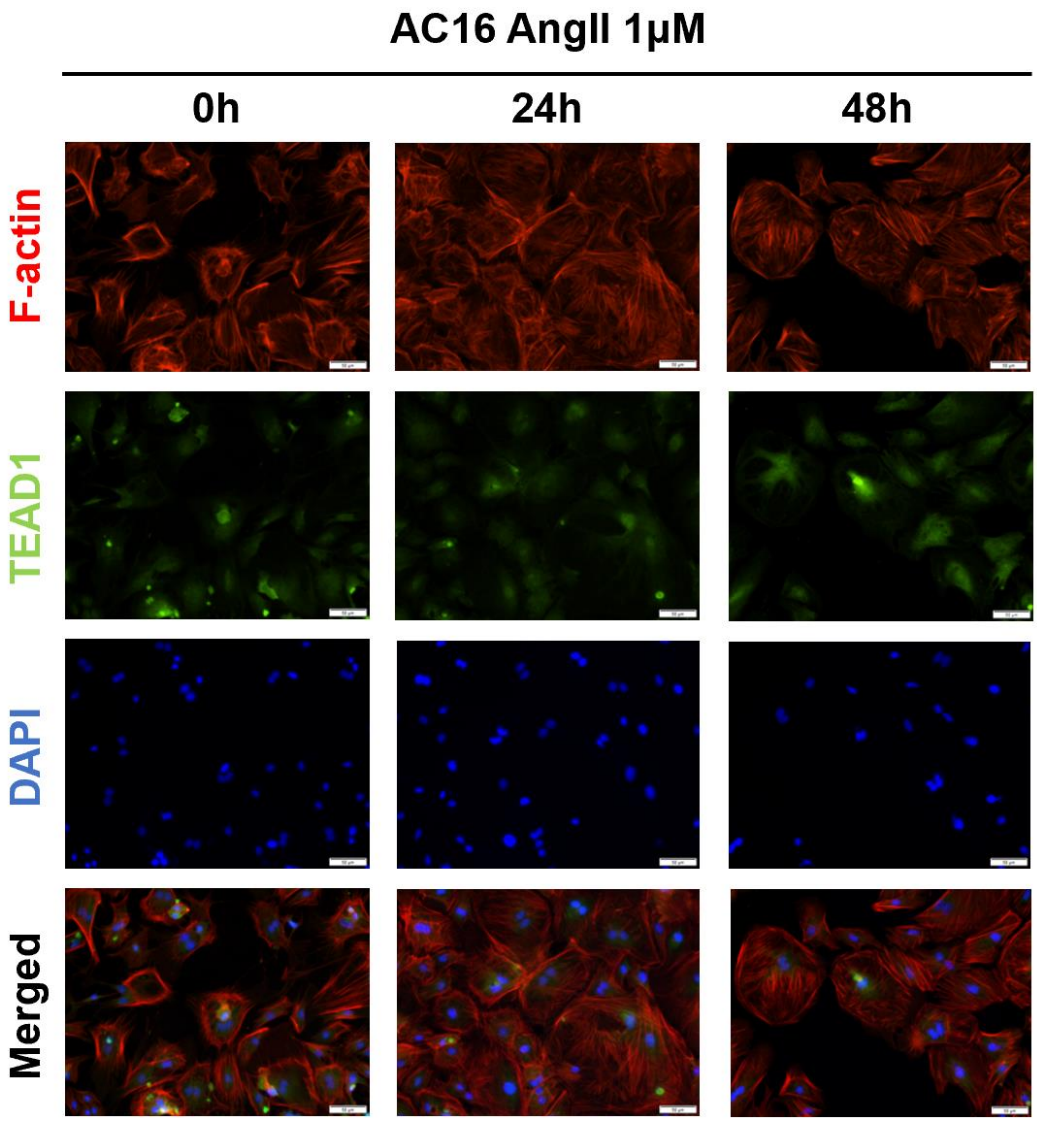

Figure S2. TEAD1 localization in the nucleus of AC16 cells. AC16 cells stained by F-actin (red) and TEAD1(green) in response to angiotensin II (1 μM) for 0h, 24h, and 48 h, respectively. The nuclei were stained with DAPI (blue). n = 3 per group; scale bar, 50 μm.

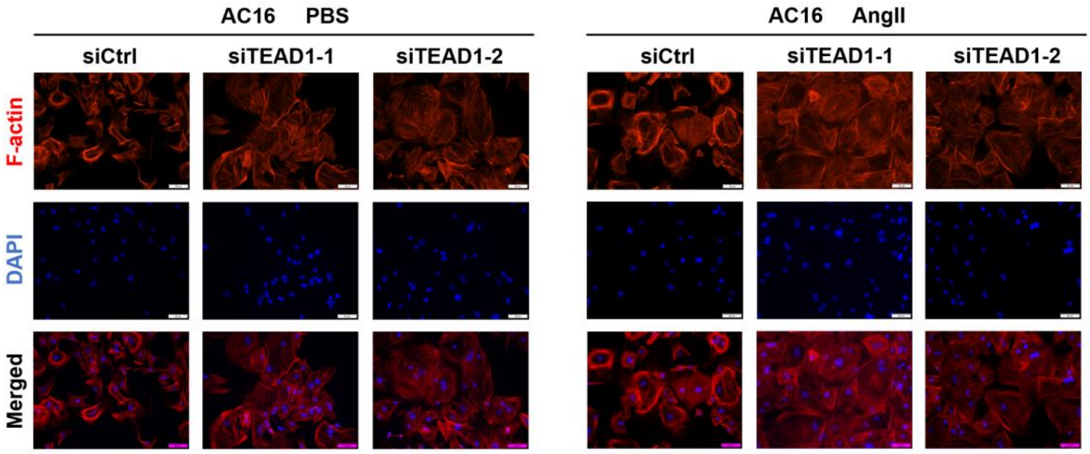

Figure S3. Representative immunofluorescence images of F-actin (red) staining of AC16 cells infected with siTEAD1-1, siTEAD1-2 or siControl. The nuclei were stained with DAPI (blue). Scale bar, 50 μm.

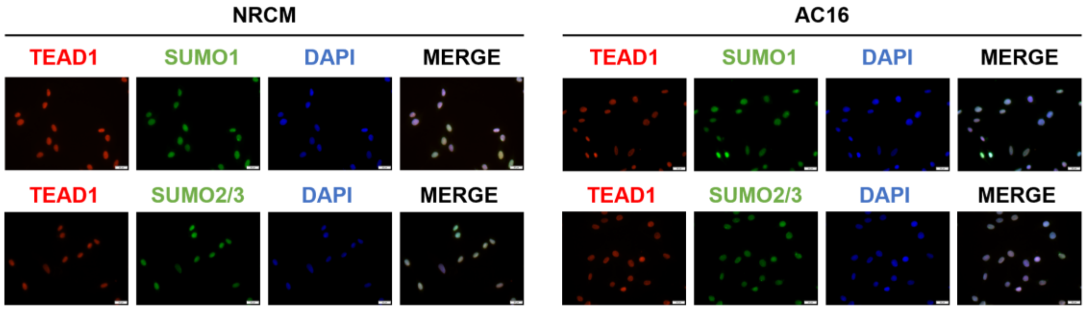

Figure S4. Representative immunofluorescence images of endogenous SUMO1(green) or SUMO2/3(green) were co-localized with endogenous TEAD1(red) in NRCMs and AC16 cells respectively. The nuclei were stained with DAPI (blue). Scale bar, 20 μm.

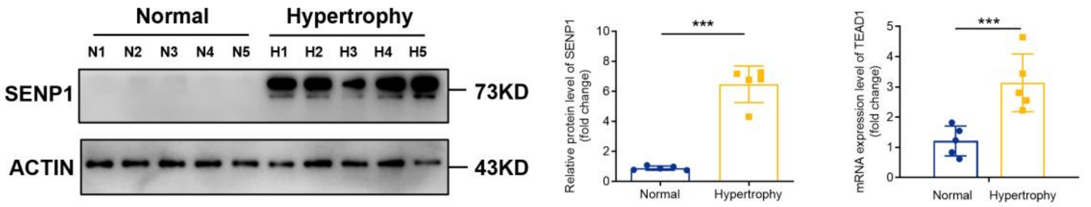

Figure S5. Representative western blots and RT-qPCR quantitative results of SENP1 protein levels in human heart tissue samples from normal donor and heart failure patients. n=5 per group.

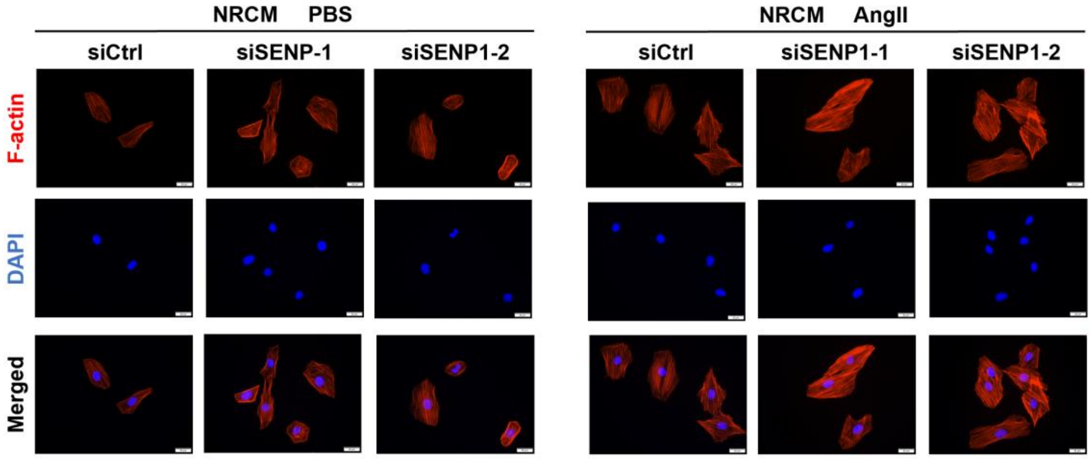

Figure S6. Representative immunofluorescence images of F-actin (red) staining of NRCMs infected with siSENP1-1, siSENP1-2 or siControl. The nuclei were stained with DAPI (blue). Scale bar, 50 μm.

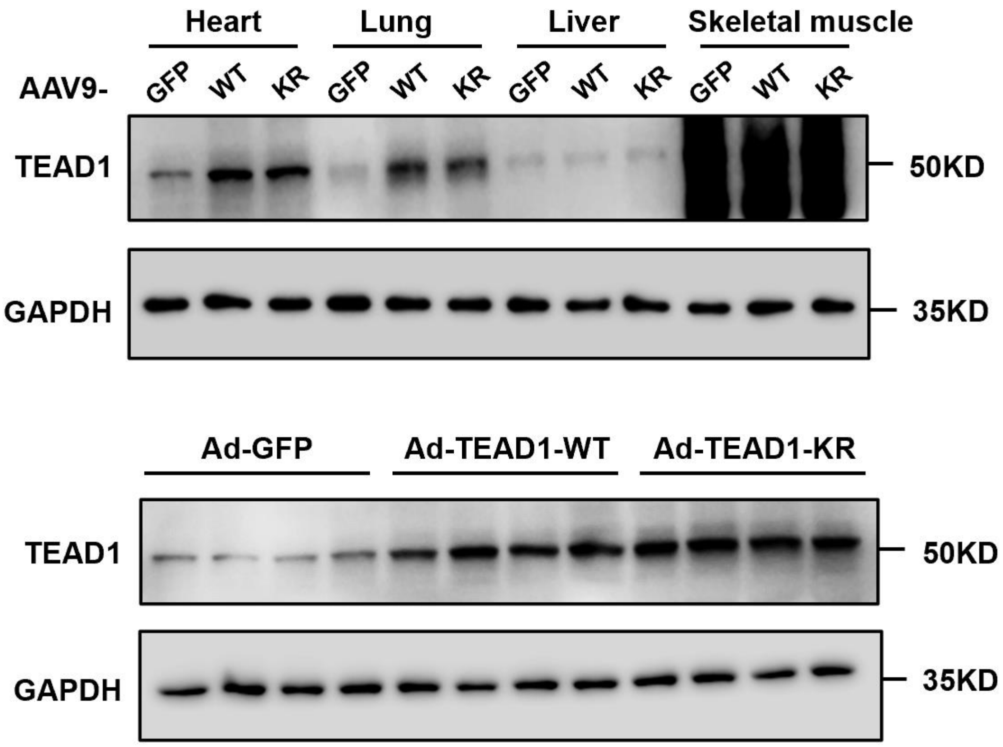

Figure S7. (A) Representative immunoblotting of TEAD1 protein expression in the heart, lung, liver, and skeletal muscle from Ad-GFP, Ad-TEAD1-WT and Ad-TEAD1-KR mice (n=3). (B) Representative immunoblotting of TEAD1 protein in the heart from Ad-GFP, Ad-TEAD1-WT and Ad-TEAD1-KR mice (n=4).

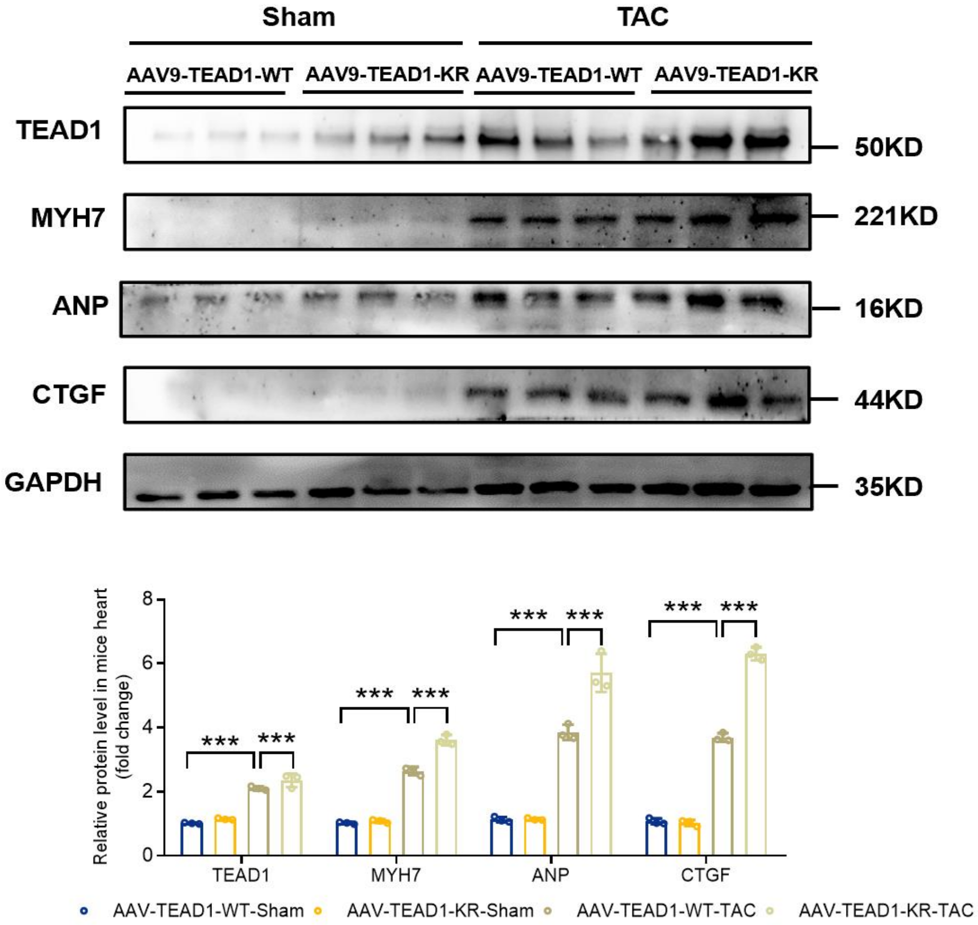

Figure S8. Representative western blots and quantitative results of hypertrophy and fibrosis marker genes in heart tissues from AAV-GFP, AAV-TEAD1-WT, and AAV-TEAD1 K177R groups at 4 weeks after sham or TAC surgery, n =3 mice per group.

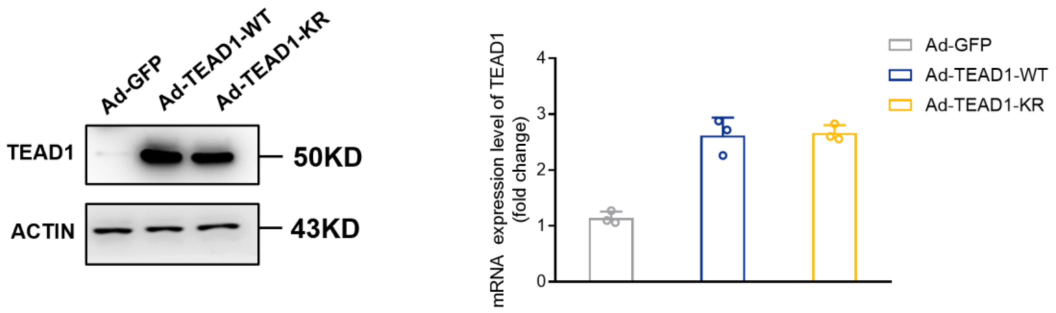

Figure S9. Representative western blots and RT-qPCR results of TEAD1 in NRCMs infected with Ad-GFP, Ad-TEAD1-WT, and Ad-TEAD1-KR. n = 3 per group.

Table S1.LC/MS (mass spectrometry) analysis in HEK293T cells s to identify the specific target of TEAD1.

Number	Gene Name	Description
1	PARP1	Poly [ADP-ribose] polymerase 1 OS
2	SSBP1	Single-stranded DNA-binding protein, mitochondrial
3	OTUD4	OTU domain-containing protein 4
4	PPHLN1	Periphilin-1
5	TRIM21	E3 ubiquitin-protein ligase TRIM21
6	SUMO1	Small ubiquitin-related modifier 1
7	CHD2	Chromodomain-helicase-DNA-binding protein 2
8	SAFB1	Scaffold attachment factor B1
9	YBX1	Y-box-binding protein 1
10	VIM	Vimentin

Table S2. Prediction of SUMO modification site and SUMO-interaction motif in JASSA (Joint Analyzer of SUMOylation site and SIMs)

Position K	Sequence	Best PS	Consensus direct			Consensus Inverted		
			Type	PSd	DB Hit	Type	PSi	DB Hit
K65	RRKIILSDEG KMYGRNELI AR	Low	None	None		Consensus inv	Low	
K78	GRNELIARYI KLRTGKTRT RK	None	None	None		None	None	
K83	IARYIKLRTG KTRTRKQVS SH	None	None	None		None	None	
K115	FHSKLKDQT AKDKALQH MAAM	None	None	None		None	None	
K173	TGQPGSSQ DVKPFVQQA YPIQ	Low	None	None		Consensus inv	Low	
K270	RQIYDKFPE KKGGLKELF GKG	None	None	None		Weak consensus inv	None	
K274	DKFPEKKGG LKELFGKGP QNA	None	None	None		None	None	
K336	VCSFGKQVV EKVETEYAR FEN	None	None	None		None	None	
K370	EYMINFIHKL KHLPEKYM MNS	None	None	None		None	None	
K425	GAQHHIYRL VKD---------	None	None	None		None	None	

Table S3. Prediction of SUMO modification site and SUMO-interaction motif in SUMOplot™ Analysis Program | Abcepta

No.	Position	Group	Score
1	K173	GSSQD VKPF VQQAY	0.58
2	K279	LKELF GKGP QNAFF	0.57
3	K336	GKQVV EKVE TEYAR	0.5
4	K265	IRQIY DKFP EKKGG	0.39
5	K269	YDKFP EKKG GLKEL	0.33
6	K270	DKFPE KKGG LKELF	0.31

Table S4. Prediction of SUMO modification site and SUMO-interaction motif in SUMOsp2.0 software

ID	Position	Peptide	Score	Cut-off	Type	Source	PPI
TEAD 1	333-337	VCSFGKQ VVEKVET EYARF	0.959	0.85	SUMO interacti on	Pred.	PPI
TEAD 1	420-424	EHGAQH HIYRLVKD *****	0.8541	0.85	SUMO interacti on	Pred.	PPI
TEAD 1	173	PGSSQDV KPFVQQA Y	0.8535	0.72	Sumoyla tion	Pred.	PPI
TEAD 1	336	FGKQVVE KVETEYA R	0.81	0.72	Sumoyla tion	Pred.	PPI
TEAD 1	289	QNAFFLV KFWADLN C	0.7717	0.72	Sumoyla tion	Pred.	PPI

Table S5. Primers for qPCR

Gene Name	Forward	Reverse
Mouse TEAD1	TCCGCTTTCCTTGAACA GCAGAG	GGGTCACTGTAAGAATG GTTGGC
Mouse TEAD2	AAGGTCTGCTCCTTTGG CAAGC	CTGACGGAGCTTGTGC AGGAAA
Mouse TEAD3	ATCGTCTCTGCCAGCGT TCTAC	CTGAGAAGGTCCAGGC TGTTGT
Mouse TEAD4	GCTCTGGATGTTGGAGT TCTCG	TTGGGCTTGACTGGCT GATGTG
Mouse ANKRD1	GCTTAGAAGGACACTTG GCGATC	GACATCTGCGTTTCCTC CACGA
Mouse CYR61	GTGAAGTGCGTCCTTGT GGACA	CTTGACACTGGAGCATC CTGCA
Mouse CTGF	TGCGAAGCTGACCTGGA GGAAA	CCGCAGAACTTAGCCCT GTATG
Mouse MYH6	GCTGGAAGATGAGTGCT CAGAG	CCAGCCATCTCCTCTGT TAGGT
Mouse MYH7	GCTGGAAGATGAGTGCT CAGAG	TCCAAACCAGCCATCTC CTCTG
Mouse ANP	TACAGTGCGGTGTCCAA CACAG	TGCTTCCTCAGTCTGCT CACTC
Mouse BNP	TCCTAGCCAGTCTCCAG AGCAA	GGTCCTTCAAGAGCTGT CTCTG
Mouse SENP1	CGTTCTTCCAGGCAGAG CTATG	GCTGTAGTGCCAATGCT TTCTGC
Mouse NQO1	AGGATGGGAGGTACTCG AATC	AGGCGTCCTTCCTTATA TGCTA
Mouse NRF2	CTGAACTCCTGGACGG GACTA	CGGTGGGTCTCCGTAA ATGG
Mouse HMOX1	GGTGATGGCTTCCTTGT ACC	AGTGAGGCCCATACCA GAAG

Mouse GAPDH	CATCACTGCCACCCAGAAGACTG	ATGCCAGTGAGCTTCCCGTTCAG
Rat MYH6	TCAAACTGGAGCTGGATGAC	GTATTCATTGGCCTGGTCCT
Rat MYH7	GAGGAGAGGGCGGACATT	ACTCTTCATTCAGGCCCTTG
Rat ANP	CACAGATCTGATGGATTTCAAGA	CCTCATCTTCTACCGGCATC
Rat BNP	GTCAGTCGCTTGGGCTGT	CAGAGCTGGGGAAAGAAGAG
Rat TEAD1	TTTGTGCAGCAGGCCTACCCCATC	GGCGAAGCTTGGTTGTGCCAATGGA
Rat TEAD2	AGCTGAAGGACCAAGTCTC	AAGGTGCAGAGATGAGCTG
Rat TEAD3	TGTCTTCTCCACCTCAAGG	AAGGGCTTGATGTCCTGAG
Rat TEAD4	AAGTTGAGACAGAGTTTGCTC	TCATGTACTCACAGAGTGGG
Rat CTGF	GGAAATGCTGTGAGGAGTGG	TGGCTCGCATCATAGTTGGG
Rat CYR61	AGAGGCTTCCTGTCTTTGGC	CTCGTGTGGAGATGCCAGTT
Rat ANKRD1	TAGAGGAGCTGGTAACGGGC	AGGTTCACACTGTTGGCTGG
Rat COL1A1	GACATGTTCAGCTTTGTGGACCC	AGGGACCCTTAGGCCATTGTGTA
Rat COL1A3	TTTGGCACAGCAGTCCAATGTA	GACAGATCCCGAGTCGCAGA
Rat GAPDH	GATGACATCAAGAAGGTGGTGA	ACCCTGTTGCTGTAGCCATATTC
Human HMOX1	CCAGGCAGAGAATGCTGAGTTC	AAGACTGGGCTCTCCTTGTTGC
Human NQO1	CCTGCCATTCTGAAAGGCTGGT	GTGGTGATGGAAAGCACTGCCT

Human NRF2	CACATCCAGTCAGAAACCAGTGG	GGAATGTCTGCGCCAAAAGCTG
Human KEAP1	CAACTTCGCTGAGCAGATTGGC	TGATGAGGGTCACCAGTTGGCA
Human TEAD1	CCTGGCTATCTATCCACCATGTG	TTCTGGTCCTCGTCTTGCCTGT
Human TEAD2	CCGCTACATCAAGCTGAGAACG	GGTTGCCATTGTCTGGAAAGCC
Human TEAD3	AGGCAGTAGATGTGCGCCAGAT	TCCTGGATGGTGCTGTTGAGGT
Human TEAD4	GAAGGTCTGCTCTTTCGGCAAG	GAGGTGCTTGAGCTTGTGGATG
Human CTGF	CTTGCGAAGCTGACCTGGAAGA	CCGTCGGTACATACTCCACAGA
Human CYR61	GGAAAAGGCAGCTCACTGAAGC	GGAGATACCAGTTCCACAGGTC
Human ANKRD1	CGACTCCTGATTATGTATGGCGC	GCTTTGGTTCCATTCTGCCAGTG
Human ACTIN	CACCATTGGCAATGAGCGGTTC	AGGTCTTTGCGGATGTCCACGT
Human ANP	ACAATGCCGTGTCCAACGCAGA	CTTCATTCGGCTCACTGAGCAC
Human BNP	TCTGGCTGCTTTGGGAGGAAGA	CCTTGTGGAATCAGAAGCAGGTG
Human MYH7	GGAGTTCACACGCCTCAAAGAG	TCCTCAGCATCTGCCAGGTTGT
Human MYH6	GGAAGACAAGGTCAACAGCCTG	TCCAGTTTCCGCTTTGCTCGCT
Human GAPHD	GTCTCCTCTGACTTCAACAGCG	ACCACCCTGTTGCTGTAGCCAA
