# Supplementary material for: SUMOylation of TEAD1 Modulates the Mechanism of Pathological Cardiac Hypertrophy
Source: Adv Sci (Weinh). 2024 Jan 15;11(12):2305677. doi: 10.1002/advs.202305677 (PMC10966521; doi:10.1002/advs.202305677)
Supplement: Supplementary file 2 — Supporting Information [file ADVS-11-2305677-s002.pdf]

ADVANCED SCIENCE
Open Access

Supporting Information

for *Adv. Sci.*, DOI 10.1002/advs.202305677

SUMOylation of TEAD1 Modulates the Mechanism of Pathological Cardiac Hypertrophy

Xin Shi, Xuening Dang, Zhenyu Huang, Yanqiao Lu, Huan Tong, Feng Liang, Fei Zhuang, Yi Li, Zhaohua Cai, Huanhuan Huo, Zhaolei Jiang, Changqing Pan, Xia Wang, Chang Gu* and Ben He**

伦理委员会审查批件

受理号：KS(Y)21240

项目名称	Lkb1 调控 VSMC 表观遗传与细胞命运参与动脉瘤形成的机制研究		
申 办 方	上海交通大学附属胸科医院		
项目类别	纵向课题	项目分期	/
申请专业	心血管内科	主要研究者	何奔
审查方式	☐会议审查 ■快速审查	审查时间	2021-03-01（材料接收日期）
审查类别	预审查		
审查文件	科研项目伦理审查申请表、课题申请书		
审查委员	徐蕾		
审查意见	1. 经本伦理委员会审查：同意该项目申报。<br>意见和建议：无。<br><br>上海市胸科医院伦理委员会<br>主任委员（签名）：[signature]<br>2021 年 3 月 5 日		

注意事项（请仔细阅读）：

1. **本批件仅表明伦理委员会同意该项目申报。若申报的项目获批，请在项目启动前重新申请伦理委员会审查；**
2. 须遵循本伦理委员会批准的方案执行，须符合 CFDA/GCP 和《赫尔辛基宣言》的原则；
3. 对已批准的研究方案、知情同意书等材料的任何修改及主要研究者更换等，须及时通知本伦理审查委员会重新审查，获得批准后执行；
4. 暂停/提前终止课题申报，请及时通知伦理委员会；
