# Supplementary material for: SUMOylation of TEAD1 Modulates the Mechanism of Pathological Cardiac Hypertrophy
Source: Adv Sci (Weinh). 2024 Jan 15;11(12):2305677. doi: 10.1002/advs.202305677 (PMC10966521; doi:10.1002/advs.202305677)
Supplement: Supplementary file 3 — Supporting Information [file ADVS-11-2305677-s003.pdf]

ADVANCED SCIENCE
Open Access

Supporting Information

for *Adv. Sci.*, DOI 10.1002/advs.202305677

SUMOylation of TEAD1 Modulates the Mechanism of Pathological Cardiac Hypertrophy

Xin Shi, Xuening Dang, Zhenyu Huang, Yanqiao Lu, Huan Tong, Feng Liang, Fei Zhuang, Yi Li, Zhaohua Cai, Huanhuan Huo, Zhaolei Jiang, Changqing Pan, Xia Wang, Chang Gu* and Ben He**

伦理委员会审查批件

受理号：KS23042

项目名称	氧化还原敏感蛋白 SENP3 介导线粒体融合/分裂失衡参与易损斑块形成的机制研究		
申 办 方	上海市胸科医院		
项目类别	纵向课题	项目分期	/
申请专业	心血管内科	主要研究者	何奔
审查方式	■会议审查　　□快速审查	审查时间	2023-11-07
审查类别	初始审查		
审查文件	具体见随附“上海市胸科医院伦理委员会审查文件清单”		
审查委员	具体见随附“上海市胸科医院伦理委员会成员及会议出席情况”		
审查意见	1. 经本伦理委员会审查：同意。<br>意见和建议：无。<br>2. 该研究进行过程中将接受伦理委员会的跟踪审查？ ■是　□否<br>跟踪审查频率为：□3 个月　□6 个月　■1 年		
	上海市胸科医院伦理委员会<br>主任委员（签名）：[illegible]<br>2023 年 11 月 9 日		

注意事项（请仔细阅读）：

1. **伦理批件仅代表项目符合伦理学原则，请申请人依据相关法律法规进行后续申报程序，获批后方能开展研究；**
2. **若研究项目或变更内容涉及人类遗传资源相关内容，请依据中华人民共和国《生物安全法》、《人类遗传资源管理条例》规定，获得相关主管部门的批准/备案成功后，方可实施；**
3. 本项临床研究应当在伦理委员批准起 1 年内实施。逾期未实施的，本批件自行废止；
4. 已批准项目须遵循本伦理委员会批准的方案执行，须符合 NMPA/GCP 和《赫尔辛基宣言》的原则；
5. 暂停/提前终止临床研究，请及时通知伦理委员会；
6. 发生严重不良事件及影响研究风险受益比的非预期事件，须及时报告本伦理委员会；
7. 对已批准的研究方案、知情同意书等材料的任何修改及主要研究者更换等，须及时通知本伦理审查委员会重新审查，获得批准后执行；
8. 发现违反方案情况须及时报告；
9. 根据伦理委员会对跟踪审查频度的意见，无论试验开始与否，请在跟踪审查日到期前 1 个月提出跟踪审查的申请。未及时提出申请的，本批件自行废止；
10. 完成临床研究，须提交结题报告供伦理委员会审查；

声明：本伦理委员会的组成及工作程序符合“药物临床试验质量管理规范”（2020 年 7 月 1 日）、“医疗器械临床试验质量管理规范”（2022 年 5 月 1 日）、“涉及人的生物医学研究伦理审查办法”（2016 年 12 月 1 日）、“涉及人的生命科学和医学研究伦理审查办法（2023 年 2 月 18 日）”中的相关要求及其所遵循的 ICH GCP 指导原则。
伦理委员会地址：上海市淮海西路 241 号；邮编：200030　联系人：陈仲林；电话：021-22200000*5341
